# Soluble CX3CL1-expressing retinal pigment epithelium cells protect rod photoreceptors in a mouse model of retinitis pigmentosa

**DOI:** 10.1186/s13287-023-03434-0

**Published:** 2023-08-21

**Authors:** Eric D. Jong, Sabiha Hacibekiroglu, Lily Guo, Evan Sawula, Biao Li, Chengjin Li, Margaret T. Ho, Molly S. Shoichet, Valerie A. Wallace, Andras Nagy

**Affiliations:** 1 Lunenfeld-Tanenbaum Research Institute, Sinai Health System, Toronto25 Orde St, 5Th Floor, Room 5-1015, Toronto, ON M5T 3H7 Canada; 2 Institute of Medical Science, Faculty of Medicine, University of Toronto, Toronto, ON Canada; 3 Institute of Biomedical Engineering, University of Toronto, Toronto, Canada; 4 Terrence Donnelly Centre for Cellular & Biomolecular Research, University of Toronto, Toronto, Canada; 5 Department of Chemical Engineering and Applied Chemistry, University of Toronto, Toronto, Canada; 6 Department of Chemistry, University of Toronto, Toronto, Canada; 7 Donald K. Johnson Eye Institute, Krembil Research Institute, University Health Network, Toronto, ON Canada; 8 Department of Laboratory Medicine and Pathobiology, University of Toronto, Toronto, ON Canada; 9 Department of Ophthalmology and Vision Sciences, University of Toronto, Toronto, ON Canada; 10 Australian Regenerative Medicine Institute, Monash University, Melbourne, VIC Australia; 11 Department of Obstetrics & Gynecology, Faculty of Medicine, University of Toronto, Toronto, ON Canada

**Keywords:** Retinal degeneration, Retinitis pigmentosa, Cell therapy, Gene therapy, Microglia activation

## Abstract

**Background:**

Retinitis pigmentosa (RP) is an inherited retinal disease that results in photoreceptor degeneration, leading to severe vision loss or blindness. Due to its genetic heterogeneity, developing a new gene therapy to correct every genetic mutation contributing to its progression is infeasible. Photoreceptor transplantation can be harnessed to restore vision; however, this approach is limited by poor cell survival and synaptic integration into the neural retina. Thus, we developed a combined cell and gene therapy that is expected to protect photoreceptors in most, if not all, cases of RP.

**Methods:**

Human embryonic stem cells (hESCs) modified with our FailSafe™ system were genetically engineered to overexpress sCX3CL1, an inhibitor of microglia activation that has been shown to preserve photoreceptor survival and function in mouse models of RP, independent of the genetic cause. These cells were differentiated into human retinal pigment epithelium (hRPE) cells and used as therapeutic cells due to their longevity and safety, both of which have been demonstrated in preclinical and clinical studies. Transgenic hRPE were delivered into the subretinal space of immunodeficient mice and the rd10 mouse model of RP to evaluate donor cell survival and retention of transgene expression. The outer nuclear layer was quantified to assess photoreceptor protection.

**Results:**

Transgenic FailSafe™ hRPE (FS-hRPE) cells can survive for at least four months in the retina of immunodeficient mice and retain transgene expression. However, these cells do not persist beyond two weeks post-injection in the retina of immunocompetent rd10 recipients, despite Cyclosporine A treatment. Nevertheless, sCX3CL1-expressing FailSafe™ hRPE cells prevented photoreceptor degeneration in a local acting manner during the duration of their presence in the subretinal space.

**Conclusions:**

Transgenic hESCs differentiate into hRPE cells and retain sCX3CL1 transgene expression both in vitro and in vivo. Moreover, hRPE cells delivered to the subretinal space of rd10 mice prevented photoreceptor degeneration in a local-acting manner, suggesting that this approach could have applications for preserving photoreceptors in specific subregions of the retina, such as the macula. Overall, our study not only reveals the potential of a combined cell and gene therapy for the treatment of RP, but also the possibility of using hRPE cells to deliver therapeutic biologics in situ to treat diseases over long-term.

**Supplementary Information:**

The online version contains supplementary material available at 10.1186/s13287-023-03434-0.

## Background

Retinitis pigmentosa (RP) is an inherited retinal degenerative disease caused by any single mutation in over 70 identified genes expressed by either photoreceptors or RPE, ultimately leading to the loss of photoreceptors [1, 2]. In 2017, Luxturna® was approved by the United States Food and Drug Administration and was anticipated to be the first possible curative treatment for RP, although it is a gene therapy that only benefits patients who express biallelic mutations in *RPE65* [3]. While gene therapy appears to be a favorable approach to prevent vision loss, developing a gene therapy for each mutated gene is inefficient [3, 4]. In addition, it carries risks of insertional mutagenesis, non-specific transduction, undesired immune responses, and genotoxicity [5–9]. Alternatively, cell therapy could be harnessed to restore vision in later stages of the disease when photoreceptors have degenerated. However, this approach carries significant hurdles, such as allogeneic cell rejection and the risk of tumorigenesis associated with pluripotent stem cell (PSC)-derived therapeutic cells that are grown in vitro [10, 11]. Although previous ground-breaking studies have demonstrated the successful transplantation of photoreceptors in mice [12, 13], more recent reports have revealed the phenomenon of material transfer between donor and host photoreceptors, which calls for a reinterpretation of previously published data [14–17]. Specifically, material transfer describes the process by which intracellular material is exchanged between donor and host photoreceptors, such as proteins, transcripts, and organelles, among others [18, 19]. Therefore, caution is required when evaluating the integration and function of donor photoreceptors in the host, as fluorescent and functional wild type proteins expressed by donor photoreceptors can be transferred to host photoreceptors. The poor survival of these cells in the subretinal space and their limited synaptic integration with the host retinal circuitry are also issues that must be addressed before cell therapy can be considered a feasible strategy [20–25].

Despite the genetic heterogeneity of RP, there is a common clinical progression of primary rod and secondary cone degeneration. This results in patients developing night blindness at some point between birth and adulthood due to rod death, which then progresses into the loss of high acuity and color vision—or even blindness—associated with cone death. While some of these mutated genes are expressed by the RPE, most are expressed by rods [2]. Since RP is a genetically diverse disease, multiple groups have identified gene-agnostic mechanisms of photoreceptor degeneration that can be targeted for its treatment, including the metabolic dysregulation of cones, oxidative stress, and microglia activation [26–33].

Microglia activation has been implicated in the pathogenesis of various neurodegenerative diseases, including Alzheimer’s and Parkinson’s disease, cerebral ischemia, and RP [33–37]. Microglia are the resident phagocytes of the retina that constantly survey the tissue for foreign stimuli, such as those released following injury [38, 39]. In the context of retinal degeneration, damage-associated molecular patterns (DAMPs) are released by dying photoreceptors, and possibly other cell types, eliciting a proinflammatory response [27, 40]. This process drives microglia activation in rodent models of RP, which is characterized by the phagocytosis of both apoptotic and healthy photoreceptors and the upregulation of proinflammatory cytokines that contribute to retinal degeneration [33, 34, 38, 41].

Thus, we explored the therapeutic potential of genetically engineered cells that overexpress a factor that inhibits microglia activation. Due to the short half-lives of recombinant proteins that can be used to prevent photoreceptor degeneration, frequent re-dosing via intraocular injections may result in severe retinal detachment, cataract, or endophthalmitis [42, 43]. While gene therapy can be harnessed to express biologics in the eye, we sought to create a novel strategy that circumvents its limitations. To do so, we aimed to develop a long-term treatment strategy that can be executed in a single intervention by delivering long-lived and quiescent cells that can secrete therapeutics to the diseased eye.

Here, FailSafe™ (FS) [44] human embryonic stem cells (hESC) were genetically engineered to overexpress soluble CX3CL1 (sCX3CL1), a ligand for the CX3CR1 receptor that is expressed by microglia. Previous studies have shown that sCX3CL1 provides a therapeutic benefit in animal models of neurodegeneration, possibly by inhibiting microglia activation [34, 35, 37, 45–49]. The transgenic cells were then differentiated into hRPE and transplanted to the subretinal space of immunodeficient mice and rd10 mice, a commonly used RP mouse model that has a point mutation in the rod cGMP-specific 3′,5′-cyclic phosphodiesterase subunit beta (*Pde6b*) [50]. In this strain, homozygous mutations in *Pde6b* result in the onset of rod photoreceptor degeneration on postnatal day (PN) 16 and cones on PN40, with the majority of rods expected to be lost by PN35.

We selected FS-hRPE as the therapeutic cell type because they can be efficiently differentiated from PSCs [51, 52] and are a long-lived and quiescent cell type [53, 54]. Additionally, they are eye-resident cells that are amenable to subretinal transplantation in animal models [52, 55–57] and have been demonstrated as safe in preclinical and early phase clinical trials [55, 58–61]. More importantly, we have described the safety and efficacy of FS for the selective elimination of proliferating donor cells in the subretinal space while leaving the host tissue unharmed [44]. Since xenograft rejection may occur in the disease model, we would expect photoreceptor protection by sCX3CL1-expressing hRPE for the duration of their survival. Taken together, our study demonstrates that sCX3CL1-expressing FS-hRPE cells can survive long-term in immunocompromised (NOD Scid Gamma, NSG) mice and can prevent photoreceptor degeneration in a mouse model of RP when injected into the subretinal space.

## Materials and methods

### Animal care

Postnatal day 14 rd10 (C57BL/6 J background) mice and eight- to 10-week-old B6 (C57BL/6 J) and NSG (NOD scid gamma/J#5557) mice were used for in vivo studies. The mice were bred at the Toronto Centre for Phenogenomics (TCP, Sinai Health System) and housed in a pathogen-free facility in micro-isolator cages on a 12-h light/dark cycle at the TCP. Both male and female mice were used for all experiments as sex was not considered as a factor. All experiments were approved by the TCP Animal Care Committee and were performed in compliance with the Animals for Research Act of Ontario and the Guidelines of the Canadian Council on Animal Care. All animal experiments adhere to the ARRIVE guidelines. At experimental endpoint, all mice were anesthetized under 4% isoflurane prior to euthanasia via cervical dislocation.

### Cell culture

H1 hESC (WiCell) were cultured on Geltrex™-coated (Gibco) plates and maintained in mTeSR Plus media (STEMCELL Technologies). Media was changed every day or every other day. Cells were passaged using ReLeSR (STEMCELL Technologies) to maintain at 80% confluency. Cultures were maintained in incubators with 5% CO_2_ at 37 °C.

### Genetic engineering of transgenic cells

The soluble CX3CL1 (sCX3CL1) construct was synthesized by Bio Basic Inc. in a pDONR vector before cloning into a vector between the inverted terminal repeats of the *piggyBac* (*pB*) transposon. The sCX3CL1 construct is encoded by the first 1008 bp of mouse CX3CL1, followed by the C-terminal addition of a FLAG and 8×-His tag, each separated by a glycine-serine linker (G_4_S). Transgenes were integrated into H1 hESC (WiCell) cells containing the FailSafe™ (FS) system using *pB* transposon-mediated transgenesis. FS hESC were transfected with FuGene® (Promega) and a combination of vectors that include a hyperactive *pB* transposase expression vector and constitutive sCX3CL1 or enhanced firefly luciferase expression cassettes in a *pB* transposon (1:1.75 of transposase to transgene expression vector). One week after transfection, FS-CX3 hESC were single-cell sorted by fluorescent activated cell sorting for the highest mCherry expressing cells to establish subclones. FS-Luc hESC subclones were derived via manual selection of single-cell-derived colonies.

### Quantitative real time—polymerase chain reaction (RT-qPCR)

RNA was extracted from cells using the GenElute™ Mammalian Total RNA Miniprep Kit (Sigma) and cDNA was synthesized using the QuantiTect Reverse Transcription Kit (Qiagen). RT-qPCR was performed using the SensiFAST SYBR No-Rox Kit (Bioline) following the manufacturer’s protocol. Primer sequences are provided in Additional file 1: Table S1.

### Western blotting

Conditioned media of hESC or hESC-derived hRPE was generated over 48 h before collection. Protein concentration was quantified with Pierce™ BCA Protein Assay Kit (cat. 23,225, ThermoFisher Scientific). For each sample, 40 µg of protein was loaded and resolved in a NuPage™ 4–12% Bis–Tris Protein Gel (10 wells, 1.5 mm) and transferred to a nitrocellulose membrane. Membranes were then blocked with 5% non-fat milk in TBS-T buffer (10 mM Tris pH 7.5, 150 mM NaCl and 0.1% Tween 20). An anti-His tag HRP-conjugated antibody (1:5000, Invitrogen, cat. P/N46-0707) was used for the detection of the sCX3CL1 construct.

### In Vitro bioluminescence assay

Human ESCs were seeded overnight in Geltrex™-coated (Gibco) 96 well black/clear bottom plates (ThermoFisher) at varying densities. Cells were washed in PBS^−/−^ (Gibco) three times before incubating in a solution that is composed of Luciferin (Perkin Elmer) and PBS^−/−^ (Gibco) (1:10) for 15 min. Bioluminescent signal was then acquired using the Infinite® 200 M200 plate reader (Tecan).

### Differentiation of hESCs into hRPE cells

RPE differentiation was carried out following an adapted protocol. Human ESC were cultured in 6-well plates and grown to over-confluence. Medium was changed to hRPE differentiation medium (replaced every 2–3 days) which contains hRPE basal medium (Knockout DMEM supplemented with 500 µg/mL penicillin/streptomycin, 1% non-essential amino acids, 1% sodium pyruvate, 2 mM GlutaMAX, and 0.1 mM β-mercaptoethanol) supplemented with 13% knockout serum replacement (KSR). Pigmented hRPE clusters appeared 3–5 weeks into differentiation and were manually picked and plated into Geltrex®-coated wells of 12-well plates. These hRPE clusters were maintained in hRPE maintenance media (RMM) which consists of hRPE basal media supplemented with 7% KSR and 5% FBS (fetal bovine serum) – media was changed every 3–4 days. Patches of hRPE from plated clusters were manually excised to purify the culture, which were maintained in RMM.

### Flow cytometry

For intracellular antigen analysis, cells were fixed and stained using a Fix and Perm kit (Invitrogen). Cells were incubated with mouse monoclonal anti-TYRP-Alexa Fluor 647 (1:400, Novus, NBP2-cat. 34720AF647) or an isotype control (1:400, Biolegend, cat. 400,155). Acquisition was performed using a BD LSRII analyzer (Becton Dickinson) and data was analyzed using FlowJo software.

### Hyaluronan methylcellulose vehicle preparation

Donor cells were resuspended in a hyaluronan (Novamatrix) and methylcellulose (Shin Etsu) hydrogel (HAMC), as previously described [62, 63]. Polymers were dissolved in Hank’s Balanced Salt Solution (HBSS) overnight at 4 °C. The gels were speedmixed, centrifuged at 16,000×*g* for 10 min, and maintained on ice until cell injection.

### Subretinal and intravitreal injections

Postnatal day 14 rd10 mice on a B6 background (cat. 004297, Jackson Laboratory) were anesthetized with isoflurane and were provided a single dose of meloxicam. Tropicamide was administered to each eye to dilate pupils. A 34-guage beveled needle attached to a Nanofil® injection system (World Precision Instruments, Sarasota, FL, USA) was used for injection. Mice either received sham injections containing 2uL of 5%/0.5% HAMC (vehicle) or 2uL of 40,000 cells suspended in 0.5%/0.5% HAMC. All mice received daily intraperitoneal injections of 10 mg/kg Cyclosporine A (Novartis, Sandimmune I.V.) beginning two days before surgery until endpoint to prevent graft rejection.

### Fundus imaging and optical coherence tomography

Mice were anesthetized with intraperitoneal injections of xylazine (20 mg/kg) and ketamine (100 mg/kg), and pupils were dilated with tropicamide solution. Fundus and optical coherence tomography (OCT) images were acquired with the Micron III digital camera system (Phoenix Research Laboratories).

### Subcutaneous injections

Eight- to 10 week-old B6 and NSG mice were anesthetized with 2% isoflurane. hRPE cells were resuspended in a 1:1 mixture of high concentration Matrigel (Corning) and high glucose Dulbecco’s Modified Eagle Medium. One million cells in 100µL were delivered into the flank subcutaneous of mice through a 23-guage needle.

### In Vivo bioluminescence imaging

Mice were anesthetized with 2% isoflurane and received intraperitoneal injections of 100 µL of 15 mg/mL solution of Luciferin (Perkin Elmer). Fifteen minutes following the injection, mice were imaged with IVIS Lumina Imager (Perkin Elmer) at an exposure of one minute; binning set to small and F/stop was set to one.

### Immunocytochemistry

hRPE were fixed in 4% PFA for 10 min at room temperature, followed by three 10 min washes with PBS for 5 min. Cells were then washed in PBS^−/−^ (Gibco) with 0.3% Triton™ X-100 (wash buffer) for five minutes, thrice, before blocking in 1% BSA, 3% FBS, 0.3% Triton X-100 in PBS^−/−^ at room temperature for 20 min. Blocking buffer was then replaced with anti-ZO1 antibody (1:100, Invitrogen, cat. 40–2200) in blocking buffer in 4˚C overnight. On the following day, wash buffer was replaced three times every five minutes before the appropriate secondary antibody (anti-rabbit IgG H&L Alexa Fluor®647, 1:400, Invitrogen, cat. A-31573) blocking buffer was applied for 1 h at room temperature. After three more washes, slides were mounted using VECTASHIELD Antifade Mounting Medium with DAPI (Vector Laboratories). Confocal images were acquired with Carl Zeiss LSM 780.

### Immunohistochemistry

Eyes were collected, fixed in 4% PFA at 4 °C overnight. PFA was then exchanged for a 30% sucrose solution at 4 °C overnight. Then, samples were embedded in O.C.T. and stored at.

-80˚C before sectioning at a thickness of 14 µm (Leica Cryostat, CM3050S). Sectioned tissue samples were stored in − 80 °C until further use.

Sections were removed from − 80 °C and air dried for 10 min at room temperature before rehydrating in TBS buffer twice for 10 min. Sections were then blocked in a TBS solution containing 5% bovine serum albumin and 0.1% Triton X-100 at room temperature for 1 h. Primary antibody solutions (mouse monoclonal anti-human nuclear antigen, 1:400, EMD Millipore, cat. MAB1281; mouse monoclonal anti-rhodopsin, 1:100, EMD Millipore, cat. MAB5316; goat polyclonal anti-OTX2, RnD Systems, BAF1979; rabbit polyclonal anti-FLAG, 1:100, Sigma, cat. F7425; goat polyclonal anti-s-opsin, Santa Cruz Biotechnology Inc., 1:1000) were prepared using the blocking solution and were then applied to sections before incubating at 4 °C overnight. On the following day, sections were washed three times with TBS for 10 min before a 2-h incubation with the appropriate secondary antibody (donkey polyclonal anti-rabbit IgG H&L Alexa Fluor®647, 1:400, Invitrogen, cat. A-31573; donkey polyclonal anti-goat IgG H&L Alexa Fluor®647, 1:400, Invitrogen, cat. A-21447; donkey polyclonal anti-mouse IgG H&L Alexa Fluor®488, 1:400, Invitrogen, cat. A-21202) for two hours at room temperature. Sections were washed three more times with TBS for 10 min before mounting with VECTASHIELD Antifade Mounting Medium with DAPI (Vector Laboratories). Confocal images were acquired with Carl Zeiss LSM 780.

### Statistical analyses

All statistical analyses were performed using GraphPad Prism 9. Unpaired Student’s *t* test was performed for comparisons between two groups. One-way ANOVA or two-way ANOVA, followed by Tukey’s post-hoc analysis was performed for multiple comparisons between groups.

## Results

### Proinflammatory environment of the degenerating retina

**Fig. 1:**
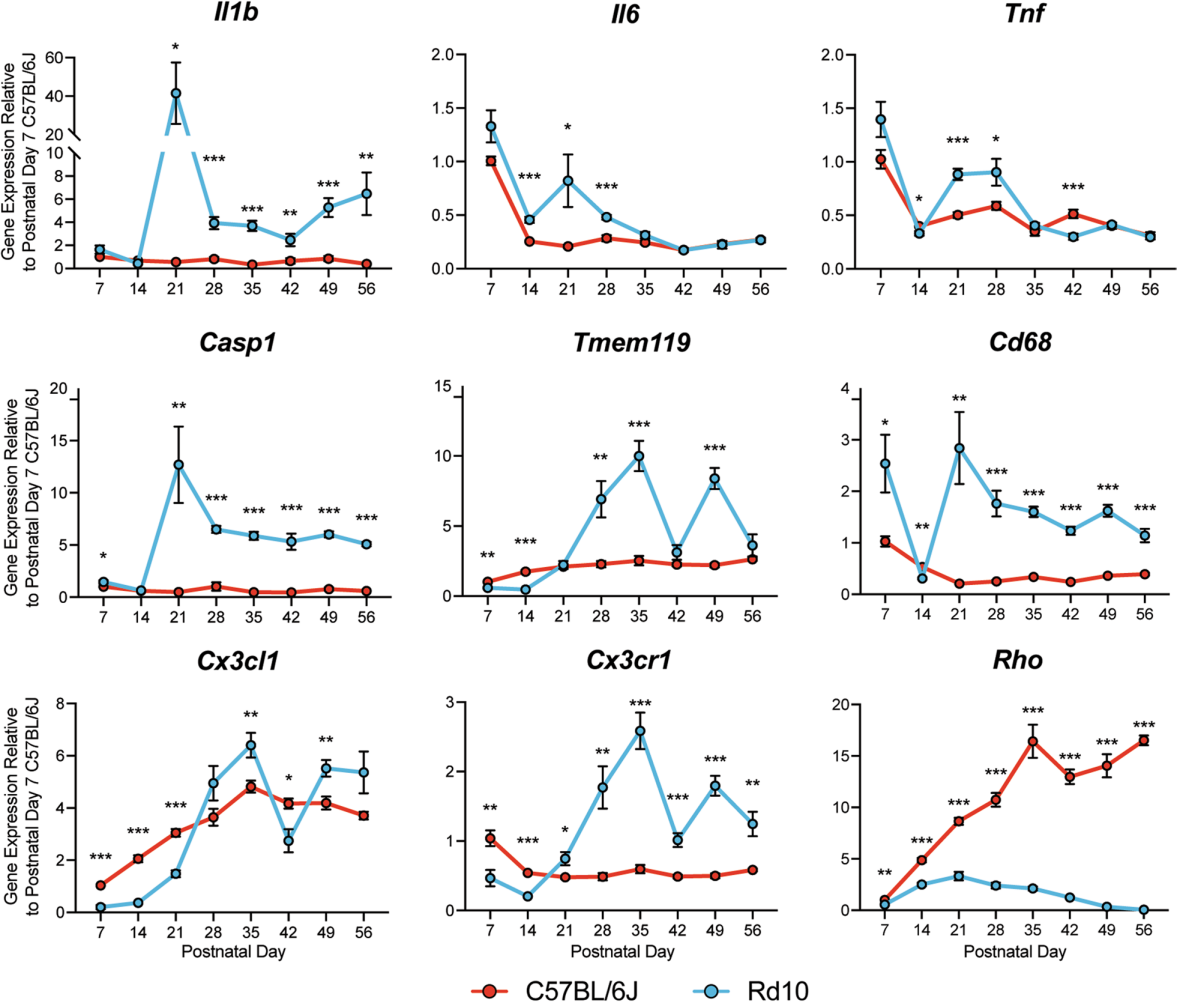
Proinflammatory genes are upregulated in the rd10 retina. RT-qPCR analysis of genes in rd10 and control C57BL/6 J retinas (*n* = 8 retinas per group). Axes represent gene expression relative to postnatal day 7 C57BL/6 J retinas. Gene expression normalized to *Gapdh*. Student’s *t*-test was performed to assess statistical significance between age groups, error bars represent SEM. *p < 0.05, **p < 0.01, ***p < 0.001, ****p < 0.0001

To develop a long-term treatment strategy for the treatment of RP, we aimed to target microglia activation as a gene-agnostic approach to preserve photoreceptors. However, we first wanted to verify the activation of microglia in the rd10 mouse model of RP. To do this, we examined the expression of proinflammatory factors in rd10 mouse retinas (C57BL/6 J background (B6)) and wild type (WT) B6 mice by RT-qPCR over one-week intervals. Although previous studies have demonstrated the expression of proinflammatory genes in the degenerating retina [27, 49, 64, 65], we also wanted to identify whether there is a connection between CX3CL1-CX3CR1 signaling and proinflammation throughout photoreceptor degeneration. Thus, we assessed the expression of proinflammatory genes [interleukin-1β (*Il1b)*, interleukin 6(*Il6)*, tumor necrosis factor alpha (*Tnfa)*, and caspase 1 (*Casp1*)] and markers of microglia activation [*Tmem119*, *Cd68*] that are known to be altered in the rd10 retina, in addition to *Cx3cl1* and *Cx3cr1*. Finally, we evaluated the expression of the rod photoreceptor marker, rhodopsin (*Rho*) (Fig. 1).

Our results show that proinflammatory and microglia activation genes, including *Cx3cr1*, were upregulated in the rd10 retina on PN21, shortly after the onset of photoreceptor degeneration. Moreover, the expression of *Il1b*, *Casp1*, and *Cd68* remain elevated in rd10 retinas, suggesting that inflammation and microglia activation persist in the degenerating retina. Conversely, *Cx3cl1* expression in the rd10 retina remained significantly lower than WT B6 retinas until 5 weeks of age (PN35). Consistent with the rd10 disease phenotype, the expression of *Rho in* rd10 retinas remained significantly lower than WT B6 retinas at all timepoints. Overall, these data confirm the proinflammatory state of the rd10 retina and reveal the upregulated expression of genes related to microglia activation throughout photoreceptor degeneration. The data also suggest a possible connection between inflammation and CX3CL1-CX3CR1 signaling in the degenerating retina. Moreover, the latent upregulation of *Cx3cl1* relative to *Cx3cr1* and other proinflammatory genes provides support for the use of exogenous sCX3CL1 as an anti-microglia activation factor for the treatment of RP.

### Development and characterization of transgenic hESCs

**Fig. 2:**
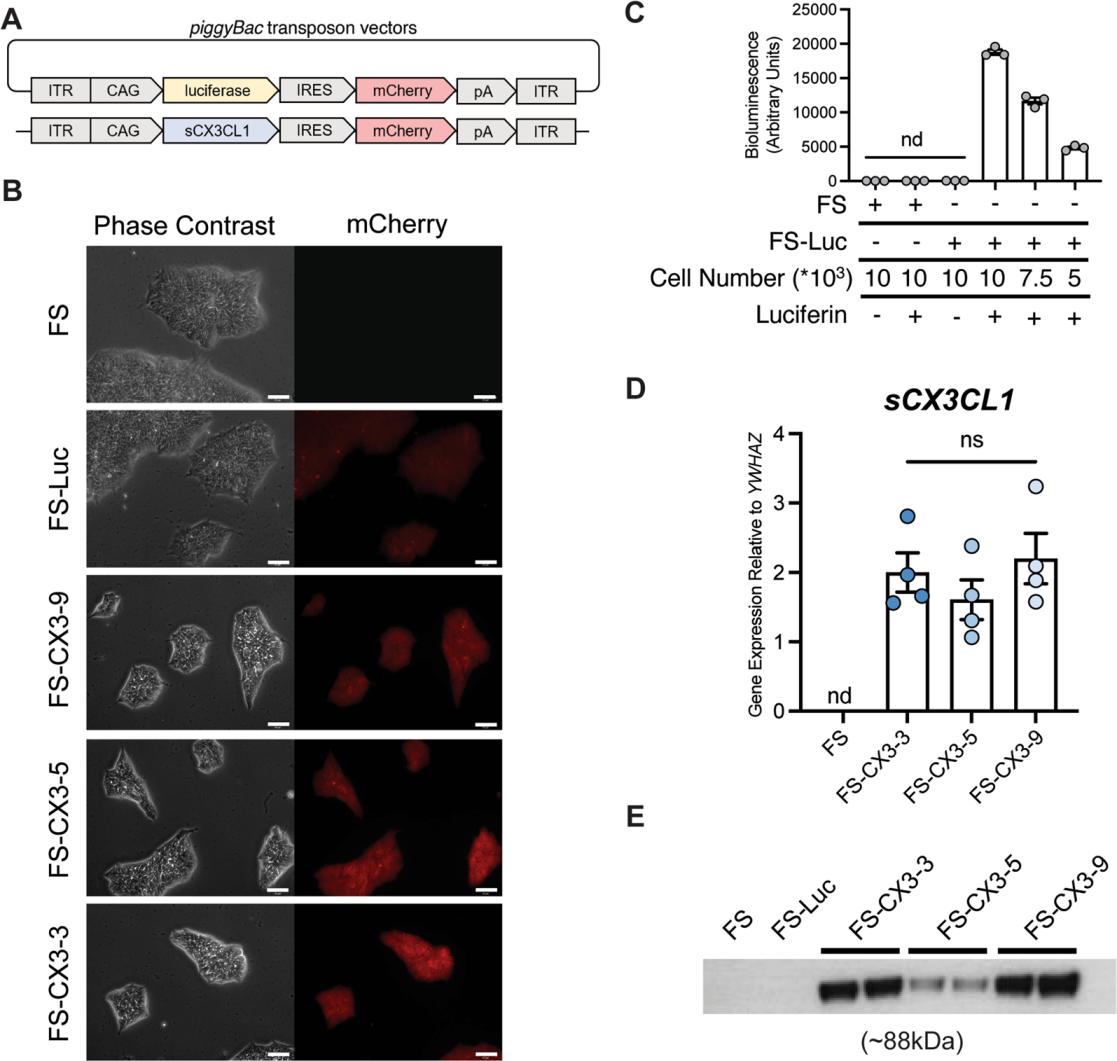
Development and characterization of transgenic FS hESC. **A** FS hESC were genetically engineered with a *piggyBac* transposon to overexpress either luciferase or sCX3CL1, each with an mCherry fluorescent reporter. **B** Phase contrast and mCherry images of parental and transgenic FS subclones that express luciferase (FS-Luc) or sCX3CL1 (FS-CX3-3, CX3-5, CX3-9) were derived, scale is 65 µm. **C** In vitro bioluminescence imaging assay of FS and luciferase-expressing FS-Luc. All comparisons are significant, ****p < 0.0001, unless stated otherwise (*n* = 3 biological replicates, each consisting of 3 technical replicates). **D** RT-qPCR analysis of sCX3CL1 RNA expression relative to housekeeping gene, *YWHAZ* (*n* = 4 biological replicates, each consisting of 3 technical replicates). Gene expression normalized to *GAPDH*. (**E**) Western blotting analysis of hESC conditioned media—two different biological replicates of media from each FS-CX3 clone were loaded. sCX3CL1 was identified by C-terminal 8×-His tag (*n* = 2–3). Full length western blot represented in Supplementary Fig. 1. One-way ANOVA followed by Tukey’s post-hoc analysis was performed on data in panels **C** and **E**. Error bars represent SD. nd, not detected; ns, not significant

Due to the risks associated with gene therapy [5–9], we aimed to protect photoreceptors with long-lived, transgenic cells that can provide a sustained release of a biologic to the diseased eye. As a general treatment strategy, we genetically modified FS hESCs to secrete a therapeutic protein and differentiated them into a cell type that is amenable to subretinal transplantation. To address issues of cell therapy safety, the FS system that was integrated into the hESCs will allow for the selective elimination of abnormal proliferating donor cells [44]. We genetically engineered FS hESCs to constitutively overexpress murine sCX3CL1 or luciferase (as a control) via *piggyBac*-mediated transgenesis (Fig. 2A). The cell lines are denoted as FS-CX3 and FS-Luc, respectively. To detect exogenous protein expression, an mCherry fluorescent reporter was linked to both transgenes by an internal ribosome entry site (Fig. 2A). C-terminal FLAG and 8×-histidine tags were also added to the sCX3CL1 construct to distinguish its expression from endogenous sCX3CL1 (Additional file 2: Fig. S1A). Subclones were derived via sparse plating of single cells (FS-Luc) or fluorescence activated cell sorting of cells that express mCherry at a high level (FS-CX3, Fig. 2B).

We next aimed to confirm the expression of luciferase and sCX3CL1 in transgenic single cell-derived FS-hESC clones. In vitro bioluminescence imaging (BLI) revealed functional luciferase activity only in the selected FS-Luc subclone when treated with luciferin (Fig. 2C). Additionally, RNA and protein expression of sCX3CL1 from three FS-CX3 subclones (FS-CX3-3, FS-CX3-5, and FS-CX3-9) were confirmed by RT-qPCR (Fig. 2D) and western blotting of conditioned media (Fig. 2E and Additional file 2: Fig. S1C), respectively. Altogether, our data show that we successfully developed genetically engineered clones of FS hESC that express either sCX3CL1 or luciferase.

### Characterization of hESC-derived hRPE

**Fig. 3:**
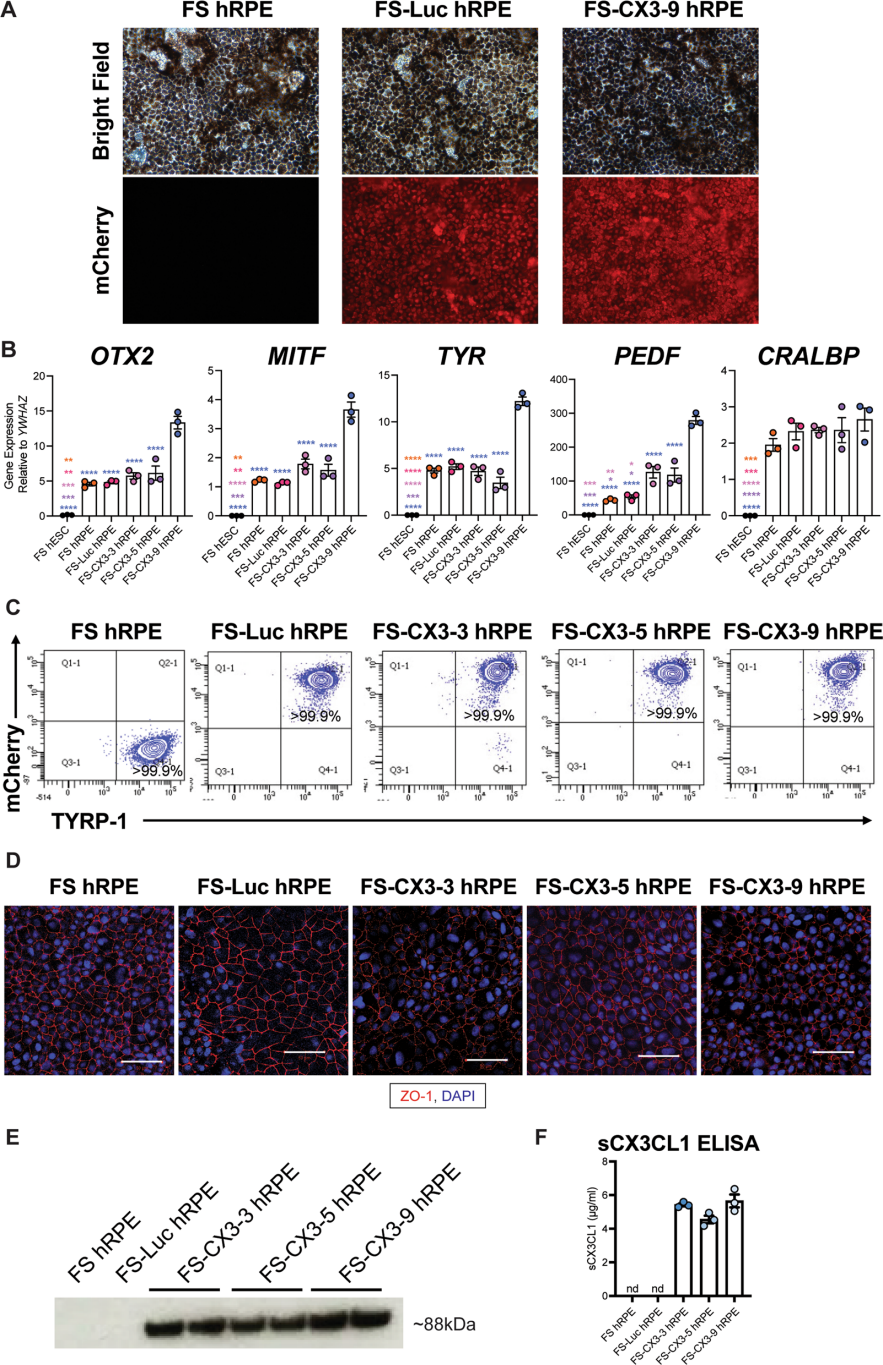
Generation and characterization of transgenic FS-hRPE cells. **A** Representative bright field and mCherry images of hESC-derived hRPE cells. Images acquired at 20× magnification. **B** RT-qPCR against a panel of RPE cell markers. Gene expression relative to *YWHAZ* and normalized to *GAPDH*. **C** Representative flow cytometry analysis plots of TYRP-1 and mCherry expression in hESC-derived hRPE (*n* = 2–3). **D** ZO-1 immunocytochemistry analysis of hESC-derived hRPE (*n* = 3–4, scale is 100 µm). **E** Western blotting analysis of hRPE conditioned media—two different biological replicates of media from each CX3 clone were loaded. sCX3CL1 was identified by C-terminal 8× -His tag (*n* = 2–3). Full length western blot represented in Additional file 4: Fig. S3. **F** Quantification of sCX3CL1 secretion by hRPE by assaying supernatant via ELISA specific to CX3CL1 (*n* = 3 biological replicates, each consisting of 2 technical replicates). One-way ANOVA and Tukey’s post-hoc analysis performed for data in panels **A** and **E.** ND, not detected; ns, not significant. **p < 0.01, ***p < 0.001, ****p < 0.0001

An ideal cell type for our combined cell and gene therapy should be quiescent, inert, and capable of long-term survival in the eye. Here, we selected hRPE cells for this strategy due to their longevity [53, 54] and safety in the subretinal space as demonstrated in both preclinical and clinical studies [55, 59–61]. Using a protocol adapted from Klimanskaya et al*.* [51], we differentiated hRPE cells from parental and transgenic FS hESCs (FS, FS-Luc, and FS-CX3 hRPE). The cells were characterized by RNA and protein expression, flow cytometry, and immunostaining. First, bright field and fluorescent microscopy showed that both parental and transgenic hRPE cells display pigmentation, however, only the transgenic hRPE cells express mCherry (Fig. 3A). RT-qPCR analysis of parental and transgenic hESC-derived hRPE revealed the expression of the RPE cell markers *OTX2*, *MITF*, *TYR*, *PEDF*, and *CRALBP* (Fig. 3B) and downregulation of the pluripotency markers *OCT4* and *NANOG* (Additional file 3: Fig. S2A). Next, while TYRP-1 was not expressed in parental and transgenic hESCs (Additional file 3: Fig. S2B), over 99.9% of mCherry^+^ transgenic hRPE cells that were differentiated from these cell lines were positive for this RPE marker by flow cytometry (Fig. 3C). Further, ZO-1 immunostaining confirmed the hexagonal morphology of hRPE cells and the presence of tight junctions (Fig. 3D). In addition, western blotting against the C-terminal 8×-His tag detected sCX3CL1 protein in the conditioned media of hRPE that are transgenic for its expression (Fig. 3E and Additional file 4: Fig. S3). An ELISA specific to sCX3CL1 was also performed on the conditioned media of the hRPE cells, which revealed no significant differences in the amount of secreted protein between the three FS-CX3 clones (Fig. 3F). In summary, we successfully differentiated hRPE from FS hESCs that are transgenic for constitutive luciferase or sCX3CL1 expression. We also show that the exogenous expression of sCX3CL1 does not affect hRPE differentiation or the expression of RPE-related genes. Importantly, the transgenic hRPE cells are pigmented, exhibit the characteristic hexagonal morphology, retain expression of the transgenes expressed at the hESC level, and express RPE-specific markers.

### Long-term survival and function of transgenic hRPE in the murine subretinal space

Before assessing the therapeutic potential of sCX3CL1-expressing hRPE cells in a mouse model of RP, we first wanted to identify whether the transgenic cells that we generated can survive in the murine subretinal space. To test this, we transplanted cells into immunodeficient NOD scid gamma (NSG) mice to eliminate the possibility of immune rejection, which would confound our results. For subretinal injections, 40,000 FS-Luc hRPE or FS-CX3-9 hRPE cells were suspended in HAMC, a hydrogel that has been demonstrated to improve cell survival post-delivery [62, 63, 66], before being delivered into both eyes of all animals.

**Fig. 4:**
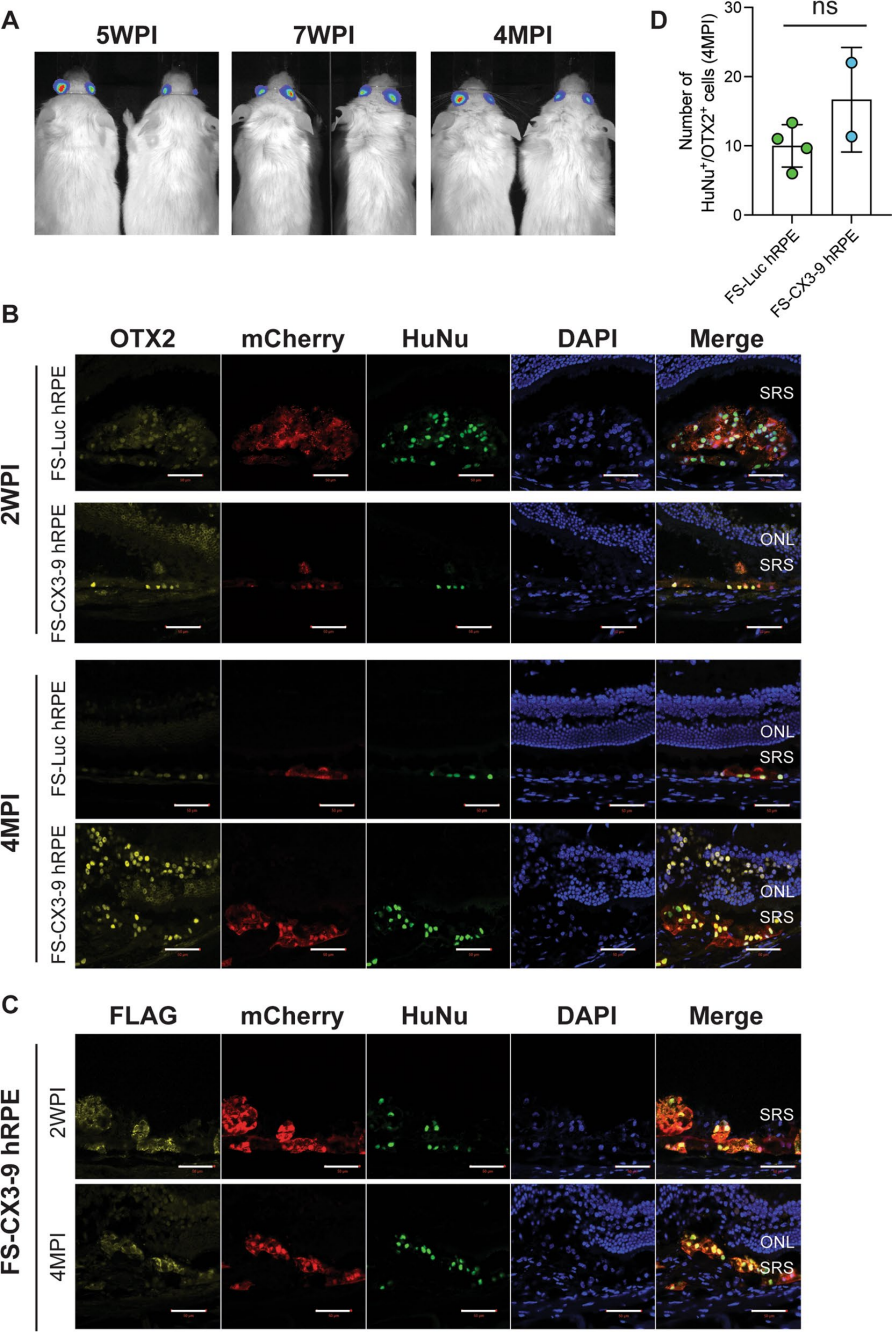
Transgenic hRPE survive and function long-term in immunodeficient mice. **A** Bioluminescent images of NSG mice treated with subretinal injections of FS-Luc hRPE. **B** OTX2 and human nuclear antigen (HuNu) co-staining of NSG retinas treated with subretinal injections of either FS-Luc hRPE or FS-CX3-9 hRPE at 2 weeks post-injection (*n* = 1 eye) and 4 months post-injection (*n* = 2–4 eyes). **C** Co-staining of FS-CX3-9 hRPE retinas for FLAG epitope (to detect exogenous sCX3CL1) and HuNu (*n* = 2 eyes). **D** Quantification of OTX2^+^/HuNu^+^ cells in the subretinal space of NSG mice at 4 months post-injection across a 200 µm distance (*n* = 2–4 eyes, each consisting of 3 technical replicates of tissue sections analyzed). Scale is 50 µm. Student’s t-test was performed to assess statistical significance. ONL, outer nuclear layer; SRS, subretinal space; WPI, weeks post-injection; MPI, months post-injection; ns, not significant

Following cell injection, optical coherence tomography (OCT) and fundus imaging of live mice at two-weeks and four-months post-injection confirmed that hRPE cells were successfully delivered into the subretinal space as indicated by deposits observed in OCT images (Additional file 5: Fig. S4). In support of this observation, BLI of FS-Luc hRPE delivered into the subretinal space of NSG mice indicated donor cell survival for at least four months (Fig. 4A). Immunostaining of retinas harvested two-weeks and four-months post-injection revealed that mCherry^+^ donor cells were positive for OTX2 and human nuclear antigen (HuNu) expression (Fig. 4B), and confirmed that FS-Luc hRPE [4 of 4 eyes] and FS-CX3-9 hRPE [2 of 2 eyes] can survive in the subretinal space for at least four months (Fig. 4B). The expression of donor-derived sCX3CL1 protein in all eyes containing mCherry^+^ FS-CX3-9 hRPE cells was also verified by anti-Flag immunohistochemistry (Fig. 4C). Further, quantification of OTX2^+^/HuNu^+^ cells across a 200 µm distance spanning the injection site or the region where donor cells were found revealed no significant difference between the survival of FS-Luc hRPE and FS-CX3-9 hRPE cells in the subretinal space of NSG mice at four-months post-injection (Fig. 4D). Finally, we did not detect tumor formation in our observations of OCT images or OTX2-HuNu stained tissues, as suggested by the absence of abnormal masses of donor cells in the subretinal space at the four-month experimental endpoint, which thus precluded the need for the use of the FailSafe™ system [44]. Overall, these data demonstrate that transgenic hRPE cells can survive long-term in the immunodeficient subretinal space and, importantly, that the expression of sCX3CL1 is retained in vivo and does not negatively impact cell survival.

### Transgenic hRPE survive short-term in the degenerating retina

**Fig. 5:**
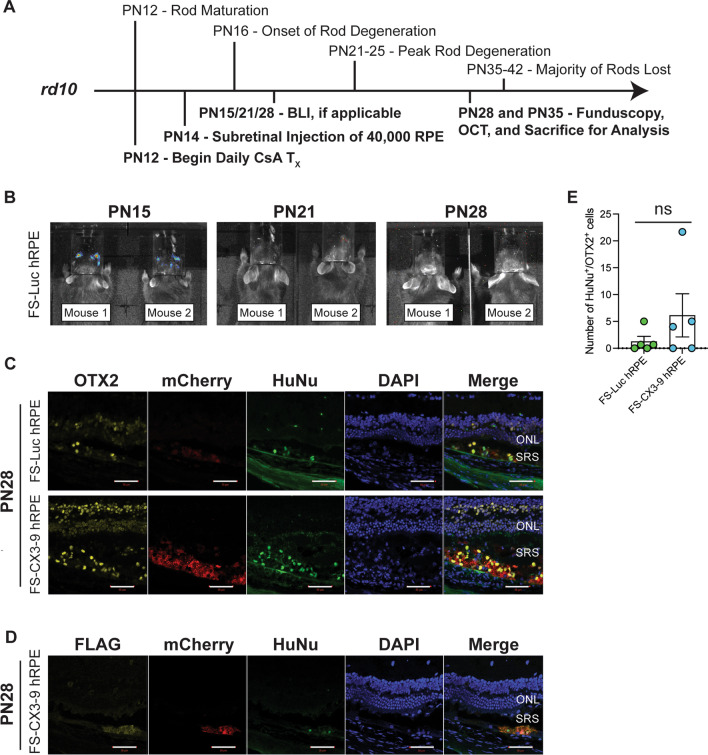
Donor hRPE survive short-term in the subretinal space of the degenerating retina. **A** Experimental timeline: Postnatal (PN) 14 Rd10 mice were treated with subretinal injections of FS-Luc or FS-CX3 hRPE cells and sacrificed at PN28 or PN35 for analysis. **B** Bioluminescence imaging of rd10 mice treated with subretinal injections of Luc hRPE. **C** Immunostaining of rd10 retinas for OTX2 and human nuclear antigen (HuNu) (*n* = 3–5 eyes). **D** Immunostaining of rd10 retinas for FLAG epitope (to detect exogenous sCX3CL1) and HuNu (*n* = 3 eyes). **E** Quantification of OTX2^+^/HuNu^+^ cells in the subretinal space of rd10 mice on PN28 across a 200 µm distance (*n* = 3–5 eyes, each consisting of 3 technical replicates of tissue sections analyzed). Scale is 50 µm. Each data point represents one biological replicate consisting of three technical replicates. Student’s *t*-test was performed to assess statistical significance. ONL, outer nuclear layer; SRS, subretinal space; WPI, weeks post-injection; ns, not significant

After confirming that transgenic hRPE cells can survive and retain transgene expression in the immunodeficient subretinal space for at least four months, we next investigated donor cell survival in the degenerating retina of the immunocompetent rd10 mouse. More importantly, we wished to explore how the disease environment may impact donor cell survival before investigating the cells’ therapeutic effect on photoreceptor preservation during active degeneration. To do so, we tracked live cells in vivo by BLI and performed immunohistochemical analyses at the experimental endpoint. Because rod photoreceptors begin to degenerate on PN16 in rd10 mice, we delivered FS-Luc hRPE or FS-CX3-9 hRPE cells into the subretinal space of both eyes on PN14, the time at which rods have matured but photoreceptor degeneration has not yet completed (Fig. 5A). To prevent xenograft rejection, rd10 mice were treated daily with Cyclosporine A (CsA), beginning two days before cells were delivered.

BLI of FS-Luc hRPE cells that were injected into the subretinal space of rd10 mice on PN14 revealed the presence of donor cells (in 6 of 6 mice) on PN15, one-day post-injection. However, BLI on PN21 revealed no signal in 5 of 6 mice and the loss of signal from all mice on PN28 (Fig. 5B). Before eyes were collected for analysis, OCT and fundoscopy on PN28 and PN35 were performed to assess if subretinal injections were successful by identifying deposits in the subretinal space or minor retinal detachments or tears (Additional file 6: Fig. S5). Despite the absence of BLI signals, immunostaining of retinas harvested on PN28 revealed the presence of mCherry^+^, OTX2^+^, and HuNu^+^ donor cells in the subretinal space of [5 of 5] FS-Luc hRPE- and [3 of 5] FS-CX3-9 hRPE-treated eyes (Fig. 5C). Thus, the lack of a BLI signal on PN28 may be attributed to an insufficient number of cells required to surpass the threshold for its detection. Conversely, mCherry^+^/OTX2^+^/HuNu^+^ donor cells were absent from all retinas harvested on PN35 (data not shown). Nonetheless, FS-CX3-9 hRPE cells stained positive for both HuNu and FLAG in PN28 retinas, demonstrating the retention of transgene expression (Fig. 5D). Quantification of OTX2^+^/HuNu^+^ donor cells across a 200 µm distance that spans the injection site or region where donor cells are found indicated no difference in the survival between the two cell lines in the rd10 subretinal space (Fig. 5E). Altogether, these data show that transgenic hRPE cells are capable of surviving in the diseased rd10 retina for up to two weeks when mice are treated with CsA. More importantly, donor cells retain transgene expression and retinal cell fate while they are alive. These results also suggest that the overexpression of sCX3CL1 has no negative impact on donor cell survival in the rd10 retina when compared to the survival of FS-Luc hRPE cells.

Since we demonstrated that both transgenic hRPE cell lines are capable of surviving for at least four months in the subretinal space of NSG mice, we next aimed to determine whether poor donor cell survival was caused by insufficient immunosuppression or the inhospitable microenvironment of the degenerating rd10 retina. To test this, FS-Luc hRPE were delivered subcutaneously into the flank of B6 mice treated with or without CsA, as well as NSG mice as a control. BLI revealed the presence of cells in NSG mice up to two weeks post-injection. However, cells were undetected in B6 mice with or without CsA treatment beyond 11 days post-injection (Additional file 7: Fig. S6). Given the similar outcome of graft survival between the two B6 groups, it is possible that the poor cell survival in the subretinal space could be attributed to insufficient immunosuppression by CsA.

### sCX3CL1-expressing hRPE delay rod photoreceptor degeneration

**Fig. 6:**
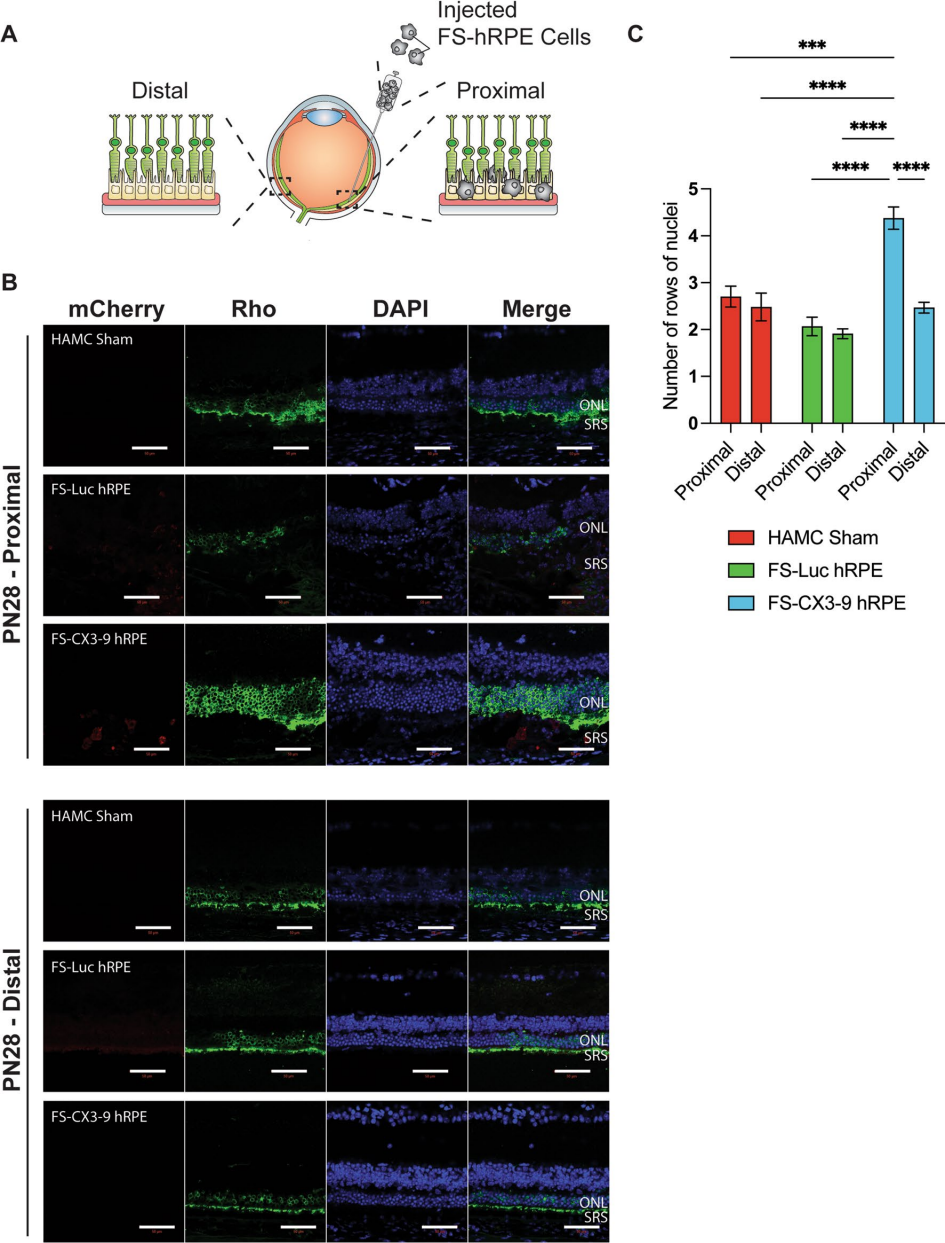
Soluble CX3CL1-expressing hRPE preserve photoreceptors in the rd10 mouse. Rd10 mice were treated on PN14 with subretinal injections of FS-Luc or FS-CX3-9 hRPE in HAMC, or HAMC alone (sham). Mice were sacrificed on PN28 for analysis. **A** Schematic for tissue analysis. Proximal region is indicated by presence of cell graft or evidence of retinal detachment. Distal region is on the opposite side of the cell graft of the same tissue section, equidistant from the optic nerve. **B** Retinas immunostained for rhodopsin. The mCherry fluorescent reporter is expressed by injected cells. (*n* = 3–7 eyes) Scale is 50 µm. **C** Quantification of rows of nuclei. (*n* = 3 eyes for sham and *n* = 5 eyes for hRPE treatment groups, each consisting of 3 technical replicates or sections analyzed). Error bars represent SEM. One-way ANOVA and Tukey’s post-hoc analysis performed for statistical analysis. All comparisons are not significant unless otherwise stated. ***p < 0.001, ****p < 0.0001. ONL, outer nuclear layer; SRS, subretinal space

To this point, we have demonstrated that transgenic hRPE cells are capable of retaining transgene expression and surviving in the murine subretinal space. Although we were only able to show that the transgenic hRPE cells survive for up to two weeks in the rd10 mouse, we have also demonstrated that these cells can survive for at least four months in NSG mice. To assess the therapeutic capacity of our approach, we next investigated whether sCX3CL1-expressing FS hRPE cells can preserve rods in the rd10 retina, given that exogenous sCX3CL1 has been shown to protect photoreceptors in the rd10 mouse model [34, 49]. To do so, we quantified photoreceptor survival as a function of ONL thickness in rd10 retinas that received subretinal injections of FS-CX3-9 hRPE, FS-Luc hRPE, or HAMC alone as the sham/vehicle control. The quantification was performed in regions with detectable mCherry^+^ donor cells. The ONL of eyes that were treated with HAMC alone and those that no longer show the presence of donor cells were quantified in regions that show evidence of minor retinal detachments or tears. More specifically, we analyzed the ONL across a 200 µm distance with the donor cell graft positioned in the center of the image in most cases (proximal) (Fig. 6A); regions of the retina that were structurally affected by retinal tears were excluded from analysis. We also quantified ONL thickness in regions opposite to the injection site and equidistant to the optic nerve of the same section (distal) to evaluate whether the treatment exerts its effect in a region where donor cells are absent (Fig. 6A). Furthermore, quantifying the ONL in the distal region of the same retinal section serves as an internal control, under the assumption that the donor cells exert their therapeutic effect in a local-acting manner.

To identify the ONL and confirm the presence of rods for quantification, we stained retinas harvested on PN28 for the expression of rhodopsin (Fig. 6B). Our results reveal that ONL thickness, expressed as rows of photoreceptor nuclei, was significantly greater in the proximal regions of the retina that were treated with FS-CX3-9 hRPE than FS-Luc hRPE and sham groups (Fig. 6C). These results suggest that the observed therapeutic effect is due to the activity of the exogenous sCX3CL1 secreted by FS-CX3-9 hRPE, and not simply the hRPE cells or HAMC alone. However, we did not observe ONL preservation in the distal regions of FS-CX3-9 hRPE-treated eyes, which may indicate a local-acting effect of the FS-CX3-9 hRPE cell treatment (Fig. 6C). Despite donor cells becoming undetected beyond PN28 in the rd10 retina, we wanted to identify whether a continued therapeutic effect of the sCX3CL1-expressing hRPE cells could still be observed at a later timepoint. As such, we examined PN35 retinas that were stained for the expression of s-opsin to reveal the ONL (Additional file 8: Fig. S7A), and the results show no significant difference in ONL thickness across all treatment groups in both proximal and distal regions (Additional file 8: Fig. S7B). More interestingly, rhodopsin staining of PN28 retinas not only verified the position of the ONL, but also revealed that the protein was present in the cell bodies of the photoreceptors and was not limited to their outer segments (Fig. 6B). This observation would suggest that while the photoreceptors are protected by exogenous sCX3CL1, the disease phenotype may not be fully ameliorated as rhodopsin remains mislocated. Ultimately, our data demonstrate that FS-CX3-9 hRPE cells can prevent photoreceptor degeneration in a local-acting manner over the duration of graft survival.

## Discussion

Here, we developed a combined cell and gene therapy as a strategy to delay photoreceptor degeneration in a mouse model of RP. First, we verified the proinflammatory state of the rd10 retina, which is consistent with reports by other groups [34, 49, 64]. Moreover, our time-course RNA expression analysis also suggests a relationship between this physiological microenvironment and CX3CL1-CX3CR1 signaling. We showed that the expression of *Cx3cr1* is upregulated along with other proinflammatory and microglia activation genes on PN21, shortly after the onset of photoreceptor degeneration in the rd10 mouse. In support of our claim, the upregulation of the CX3CL1-CX3CR1 signaling axis has been observed in other models of the injured central nervous system in which inflammation is present [67, 68]. We also revealed the latent upregulation of RNA expression of the ligand, *Cx3cl1*, relative to the markers of inflammation and microglia activation in the rd10 retina. Specifically, this was observed on PN35, when most of the rods have degenerated. On the protein level, Zieger et al*.* [65] reported lower levels of CX3CL1 in the rd10 retina compared to WT, revealing lower ligand expression in the diseased retina. More importantly, however, their study demonstrated that the soluble form of CX3CL1 is only detected by western blotting analysis in PN5, PN45, and PN60 rd10 retinas [65], indicating the absence of sCX3CL1 during which the majority of photoreceptors are degenerating [50]. Taken together, our results, along with those from studies that have demonstrated the neuroprotective effects of CX3CL1 [34, 35, 37, 49, 69], support the use of exogenous sCX3CL1 to inhibit microglia activation for the treatment of RP.

Previous reports indicated that CX3CL1 exerts a neuroprotective effect by targeting activated microglia [69, 70], which has been demonstrated in rodent models of stroke, Parkinson’s disease, and Alzheimer’s disease [35–37]. Microglia are the resident immune cells of the central nervous system, including the retina. They are responsible for antigen presentation, complement activation, and inducing inflammatory reactions. Aside from their role in retinal degeneration, they are also integral to photoreceptor maturation and maintenance [71, 72]. Following the onset of photoreceptor degeneration in animal models of RP, microglia are activated, migrate to the ONL, and transition from a ramified to amoeboid morphology [28]. The main processes that drive disease progression involve their phagocytosis of both viable and apoptotic rods, secretion of proinflammatory cytokines (such as IL-1B), and production of reactive oxygen species and other biologically active molecules, like nitric oxide [27, 29, 33]. Intriguingly, in vitro and in vivo studies have shown that exogenous CX3CL1 inhibits microglia activation and can stimulate their expression of anti-inflammatory genes, providing evidence for its mechanism of action [34, 47]. Thus, when microglia are ablated from the degenerating retina, photoreceptors are preserved as expected [33], which further justifies sCX3CL1-mediated inhibition of microglia activation as a feasible approach to the treatment of RP.

With respect to inherited retinal diseases, sCX3CL1 has been shown to protect photoreceptors in multiple mouse models of RP, including the rd10 mouse used here in our study [34, 49]. Wang et al*.* [49] revealed the promise of an AAV8-sCX3CL1 gene therapy that protected cone photoreceptors and their function over long-term in multiple mouse models of RP, but were unable to show that sCX3CL1 overexpression inhibits microglia activation. However, it is worth considering two factors of their study that have led to this outcome. First, the sCX3CL1 gene therapy was introduced to neonatal (PN0-1) pups before the retina has fully developed. It is important to note that microglia and the CX3CL1-CX3CR1 signaling axis play integral roles in the development and maturation of photoreceptors [71, 72]. Thus, the presence of supraphysiological sCX3CL1 levels before the retina has fully developed may have altered the niche’s microenvironment over long-term. Doing so increases the difficulty of determining its therapeutic mechanism, as the timing of the intervention might have also affected the microglia phenotype. Second, the authors aimed to detect the effects of microglia on cones in rd1 mice (a mouse model of rapid RP) by depleting microglia with PLX-3397 after most photoreceptors have degenerated. We argue that it would be more meaningful if the authors started PLX-3397 treatment before rod degeneration is complete. Zabel et al. [34] demonstrated that the short-term treatment of rd10 mice with intravitreal bolus injections of CX3CL1 reduces the number of infiltrating microglia in the ONL, the density of microglial phagosomes in the ONL, and microglia activation (as indicated by diminished CD68 immunopositivity). In support of the pathological role of reactive microglia in the degenerating retina, a previous study demonstrated that tamoxifen-induced ablation of retinal microglia in rd10 mice crossed with CX3CR1^CreER^ × Rosa26-flox-STOP-flox-DTA mice on PN21-23 resulted in significant histological rescue and the preservation of visual function [33]. Taken together, the sustained release of sCX3CL1 is potentially a viable long-term therapeutic strategy for the treatment of RP that acts by inhibiting microglia activation.

As an alternative to gene therapy, we aimed to protect photoreceptors by using cells as a factory to deliver a sustained supply of sCX3CL1 to the degenerating retina. Gene therapy is limited by the lack of control over transgene expression levels, as dose-dependent vector toxicity can occur in the eye [73]. With therapeutic cells, we can control the amount of exogenous sCX3CL1 delivered into the eye by modifying the number of donor cells that are delivered to the diseased site. Furthermore, the biologics released by cells in situ are of ‘clinical grade’ such that toxic contaminants are not of concern, as is the case with recombinant protein therapy. Critically, the FS system ensures the safety of the combined cell and gene therapy, whereas tumor development by endogenous cells caused by insertional mutagenesis of viral vectors cannot be controlled [44]. Overall, these factors underscore the benefits of using cells for exogenous gene expression.

In our study, human FS ESC [44] were genetically engineered to overexpress sCX3CL1 or luciferase and were then differentiated into RPE cells. The soluble form of CX3CL1, which was used here, encodes the first 336 amino acids—excluding the transmembrane and intracellular domains—like the construct used by Wang et al*.* [49]. Since the donor cells were delivered into the subretinal space, we selected the soluble form of CX3CL1, hoping that it would diffuse throughout the retina and exert its therapeutic effect on the entire tissue. We genetically engineered hESCs, as they can be differentiated into any cell type the user would deem optimal for the therapy. This allowed us to differentiate transgenic hESCs into eye-resident RPE cells, satisfying our requirement of a long-lived and quiescent cell type for this therapeutic strategy. In further support, clinical trials have reported that hRPE cells exhibit longevity, quiescence, and safety in the subretinal space of patients with diseased retinas [52, 55, 59–61].

Before assessing the therapeutic potential of this combined cell and gene therapy, we first determined whether the transgenic hRPE can survive in the murine subretinal space. BLI and histological analyses show that these cells can survive for at least 4 months, retain RPE cell fate, and maintain transgene expression in the NSG retina. However, these cells were undetectable in the rd10 retina beyond two weeks post-injection, even after immunosuppression with CsA to suppress systemic T cell activation. In order to identify whether the loss of donor cells was the result of insufficient immunosuppression, we subcutaneously delivered luciferase-expressing hRPE cells into the dorsal flank of B6 mice treated with or without CsA, as well as NSG mice as a control. We observed a lack of BLI signal 11 days post-injection in both B6 groups, suggesting it is more likely that donor cells were rejected due to insufficient immunosuppression of the host. Supporting this claim, recent studies have demonstrated the preservation of donor cells in the degenerating retina when the host is sufficiently immunosuppressed. For example, Zhu et al., [57] delivered hRPE differentiated from human induced pluripotent stem cells into the subretinal space of rd10 mice and demonstrated robust donor hRPE cell survival up to 14 days post-surgery by immunosuppressing the mice with both CsA and prednisolone. Another study demonstrated the requirement of an immunosuppressive cocktail, which also includes prednisolone, that protects up to 70% of a human hRPE cell graft over a 10-week period in a xenogeneic recipient [52]. Similarly, Ripolles-Garcia et al*.* [24] revealed that a cocktail consisting of CsA, prednisolone, and mycophenolate mofetil are required to prevent the rejection of human immature photoreceptors from the degenerating canine retina. Prednisolone is a corticosteroid that exerts its immunosuppressive effect by inhibiting microglia activation, and since we anticipated that sCX3CL1 targets microglia to elicit a therapeutic effect, we decided to exclude corticosteroids from our study as these drugs may inadvertently confound our results [74–76]. Since CsA was the only immunosuppressant used in our study, it is likely that insufficient immunosuppression, and not the microenvironment of the degenerating retina, contributed to donor cell rejection in our study.

Here, we demonstrated that FS-CX3-9 hRPE cells preserved photoreceptors in the rd10 retina for as long as they persisted in the subretinal space. In contrast to previous studies that demonstrated photoreceptor protection by non-transgenic human hRPE [56, 57], we were unable to show ONL preservation in rd10 mice by FS-Luc hRPE cells when compared to the sham-treated groups. Perhaps these differences may have been caused by the variability between different PSC lines and differentiation protocols. More strikingly, we show that only the FS-CX3-9 hRPE cells, and not the controls, are capable of preserving photoreceptors in the degenerating retina, which would suggest that the therapeutic effect is the direct result of exogenous sCX3CL1 activity. We also show that only FS-CX3-9 hRPE cells preserved photoreceptors in a local-acting manner (i.e., where the donor cells are present). This observation came as a surprise, as we expected the secreted sCX3CL1 to diffuse throughout the retina. Gregory-Evans et al. [77] reported a similar outcome after the intravitreal injection of glial cell-derived neurotrophic factor-expressing mouse embryonic stem cells (mESC) into the TgN S334ter model of RP. Despite the alternative route of administration, most of the mESC were attached to the peripheral retina and photoreceptor preservation was only detected in the regions of the ONL that were adjacent to donor cells [77]. Thus, it is plausible that the highly dense retina is not conducive to the diffusion of soluble proteins throughout the tissue and, as a result, our therapeutic strategy could be more effective in treating specific tissue regions, such as the macula.

Overall, we show that transgenic cells have the potential to be used as a factory for the sustained release of a therapeutic factor to prevent photoreceptor degeneration. While we only identified photoreceptor preservation over the duration of the FS-CX3-9 hRPE graft survival, we believe that the long-term protection of photoreceptors is possible if we can prevent xenograft rejection. Such an outcome is encouraging given the long-term survival of the transgenic cells in NSG mice alongside studies that have demonstrated xenograft tolerance with immunosuppressive cocktails [23, 24, 52, 57], as well as the success of a sCX3CL1 gene therapy that protected photoreceptors and their function over long-term [49].

## Conclusions

In this study, we differentiated transgenic FS-hESC into hRPE cells that can deliver therapeutic proteins in vivo. Using the *piggyBac* transposon gene delivery system, we overexpressed sCX3CL1 in FS-hESC and showed that the cells can differentiate into hRPE and retain transgene expression. When these cells were delivered into the subretinal space of immunodeficient mice, we demonstrated that they are capable of surviving for at least four months post-injection, while retaining transgene expression and hRPE cell fate. In the rd10 mouse model of RP, both FS-Luc and FS-CX3-9 hRPE cells are undetectable in the subretinal space beyond two weeks post-injection, despite CsA treatment. However, only the FS-CX3-9 hRPE cell treatment preserved the ONL, albeit in a local-acting manner, and the therapeutic effect was not detected beyond graft survival. Since photoreceptor preservation of the rd10 retina was not observed in the control groups, our results suggest that the therapeutic effect of FS-CX3-9 hRPE cells was afforded by sCX3CL1 overexpression. In line with this finding, our data suggest that hRPE cells can be used as a factory to deliver biologics to the retina as a general treatment strategy. However, further studies are required to establish the long-term survival of hRPE in a xenogeneic host so that the long-term therapeutic efficacy of the sCX3CL1-expressing hRPE cells can be determined, including its effect on visual function.

### Supplementary Information

**Additional file 1**. The list of PCR primers used and their corresponding sequences.

**Additional file 2**. Fig. S1 Generation and characterization of sCX3CL1-expressing hESC. **A** Schematic of sCX3CL1 construct. **B** Plots representing the sort of top 4.72% mCherry-expressing FS hESC that were transfected with the sCX3CL1 *piggyBac* expression cassette. **C** Western blotting analysis against 8×-His tag of hESC conditioned media and respective global protein stain (*n* = 2–3). Glycine, G; Serine, S; FLAG, FLAG tag; Histidine, His.

**Additional file 3**. Fig. S2. Characterization of the expression of pluripotent stem cell markers in hRPE cells and the expression of a mature hRPE marker in hESC. **A** RT-qPCR against a panel of pluripotency markers (*OCT4, NANOG*). Gene expression normalized to *GAPDH* and expressed as relative to *YWHAZ*. **B** Representative flow cytometry analysis plots of TYRP-1 and mCherry expression in parental and transgenic hESC. Data analyzed by one-way ANOVA and Tukey’s post-hoc analysis. ****p < 0.0001. Fig. S3. Full-length western blotting of hRPE conditioned media. Western blotting analysis against 8×-His tag of hESC conditioned media and respective global protein stain (*n* = 2–3).

**Additional file 4**. Fig. S3. Full-length western blotting of hRPE conditioned media. Western blotting analysis against 8×-His tag of hESC conditioned media and respective global protein stain (*n* = 2–3).

**Additional file 5**. Fig. S4 In vivo imaging of NSG mice. Representative fundus and optical coherence tomography images (OCT) of **A** FS-Luc hRPE- or **B** FS-CX3-9 hRPE-treated NSG retinas. WPI, weeks post-injection; MPI, months post-injection.

**Additional file 6**. Fig. S5 In vivo imaging of rd10 mice. Representative fundus and optical coherence tomography images (OCT) of **A** FS-Luc hRPE- or **B** FS-CX3-9 hRPE-treated rd10 retinas. PN, postnatal.

**Additional file 7**. Fig. S6 Human hRPE delivered to flank subcutaneous are rejected in B6 mice. Representative bioluminescence imaging of mice treated with subcutaneous injections of FS-Luc hRPE at indicated timepoints. Cyclosporine A, CsA. (*n* = 3–4 mice).

**Additional file 8**. Fig. S7 Photoreceptor preservation in the rd10 mouse is not observed on PN35. Rd10 mice were treated on PN14, prior to the onset of rod degeneration, with subretinal injections of FS-Luc or FS-CX3-9 hRPE in HAMC, or HAMC alone (sham). Mice were sacrificed on PN35 for analysis. **A** Retinas immunostained for s-opsin. mCherry fluorescent reporter is expressed by injected cells. **B** Quantification of rows of nuclei (*n* = 3–4 eyes, each consisting of 3 technical replicates or tissue sections analyzed). Scale is 50 µm. One-way ANOVA and Tukey’s post-hoc analysis performed for statistical analysis. All comparisons are not statistically significant. ONL, outer nuclear layer; SRS, subretinal space.

## Data Availability

The data used and analyzed in the study are available.

## Abbreviations

BLI: Bioluminescence imaging
CsA: Cyclosporine A
DAMP: Damage-associated molecular patterns
FS™: FailSafe™
HAMC: Hyaluronan methyl cellulose
hESC: Human embryonic stem cells
hRPE: Human RPE
HuNu: Human nuclear antigen
mESC: Mouse embryonic stem cells
NSG: NOD scid gamma
OCT: Optical coherence tomography
ONL: Outer nuclear layer
PN: Postnatal
PSC: Pluripotent stem cells
RP: Retinitis pigmentosa
RPE: Retinal pigment epithelium
sCX3CL1: Soluble CX3CL1
WT: Wild type
