# Supplementary material for: Soluble CX3CL1-expressing retinal pigment epithelium cells protect rod photoreceptors in a mouse model of retinitis pigmentosa
Source: Stem Cell Res Ther. 2023 Aug 21;14:212. doi: 10.1186/s13287-023-03434-0 (PMC10441732; doi:10.1186/s13287-023-03434-0)
Supplement: Supplementary file 1 — Additional file 1. The list of PCR primers used and their corresponding sequences. [file 13287_2023_3434_MOESM1_ESM.docx]

| Primer | Sequence (5'-3') |
| --- | --- |
| sCX3CL1-F | TGCGACAAGATGACCTCACG |
| sCX3CL1-R | GGCGTCTTGGACCCATTTCT |
| hOCT4_F | CTGTCTCCGTCACCACTCTG |
| hOCT4_R | TGTGTTCCCAATTCCTTCCTTAG |
| hNANOG_F | CCCTCCTCCCATCCCTCATAG |
| hNANOG_R | TCGCTGATTAGGCTCCAACC |
| hMITF_F | TTGTCCATCTGCCTCTGAGTAG |
| hMITF_R | CCTATGTATGACCAGGTTGCTTG |
| hOTX2_F | ACCTTGAACTCCACCTCTGC |
| hOTX2_R | GCTTCTCTTCTCTGACTCTCTTTG |
| hTYR_F | GTGTAGCCTTCTTCCAACTCAG |
| hTYR_R | GTTCCTCATTACCAAATAGCATCC |
| hPEDF_F | TATCACCTTAACCAGCCTTTCATC |
| hPEDF_R | GGGTCCAGAATCTTGCCAATG |
| hCRALBP_F | AGATCTCAGGAAGATGGTGGAC |
| hCRALBP_R | GAAGTGGATGGCTTTGAACC |
| hYWHAZ_F | CCGCCAGGACAAACCAGTAT |
| hYWHAZ_R | ACTTTTGGTACATTGTGGCTTCAA |
| hGAPDH_F | CATGAGAAGTATGACAACAGCCT |
| hGAPDH_R | AGTCCTTCCACGATACCAAAGT |
| mCx3cl1-F | TGCGACAAGATGACCTCACG |
| mCx3cl1-R | GGCGTCTTGGACCCATTTCT |
| mCx3cr1-F | ACCTCCTTCCCTGAACTGGA |
| mCx3cr1-R | CAGACCGAACGTGAAGACGA |
| mIl1b-F | ATGCCACCTTTTGACAGTGA |
| mIl1b-R | GTGCTGCTGCGAGATTTGAA |
| mIl6-F | TAGTCCTTCCTACCCCAATTTCC |
| mIl6-R | TTGGTCCTTAGCCACTCCTTC |
| mTnfa-F2 | CCCTCACACTCAGATCATCTTCT |
| mTnfa-R2 | GCTACGACGTGGGCTACAG |
| mCasp1-F3 | GGACCCTCAAGTTTTGCCCT |
| mCasp1-R3 | GCTCCAACCCTCGGAGAAAG |
| mCd68-F1 | GCTAGGACCGCTTATAGCCC |
| mCd68-R1 | GGATGGCAGGAGAGTAACGG |
| mTmem119-F1 | CCTACTCTGTGTCACTCCCG |
| mTmem119-R1 | CACGTACTGCCGGAAGAAATC |
| mRho-F2 | CTTCACCTGGATCATGGCGTT |
| mRho-R2 | TTCGTTGTTGACCTCAGGCTTG |
| mGapdh-F | CAAGGTCATCCATGACAACTTG |
| mGapdh-R | GTCCACCACCCTGTTGCTGTAG |
| mHprt-F | GGCCAGACTTTGTTGGATTTG |
| mHprt-R | TGCGCTCATCTTAGGCTTTGT |
