# Supplementary figures and images for: Soluble CX3CL1-expressing retinal pigment epithelium cells protect rod photoreceptors in a mouse model of retinitis pigmentosa
Source: Stem Cell Res Ther. 2023 Aug 21;14:212. doi: 10.1186/s13287-023-03434-0 (PMC10441732; doi:10.1186/s13287-023-03434-0)

**A**

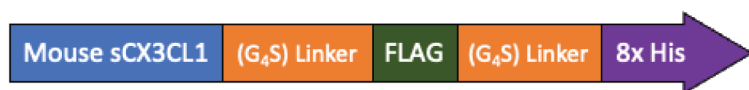

**B**

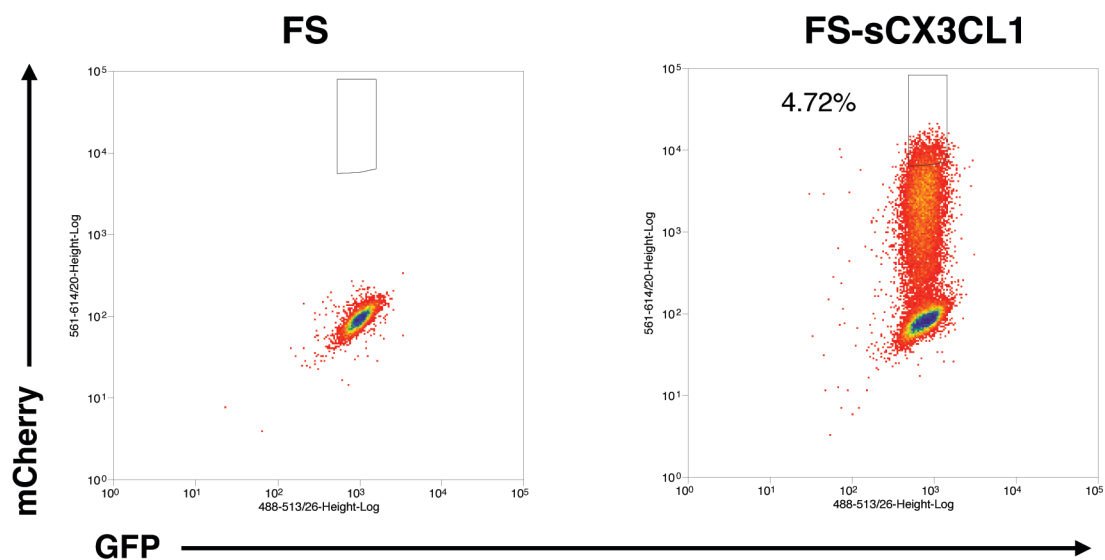

**C**

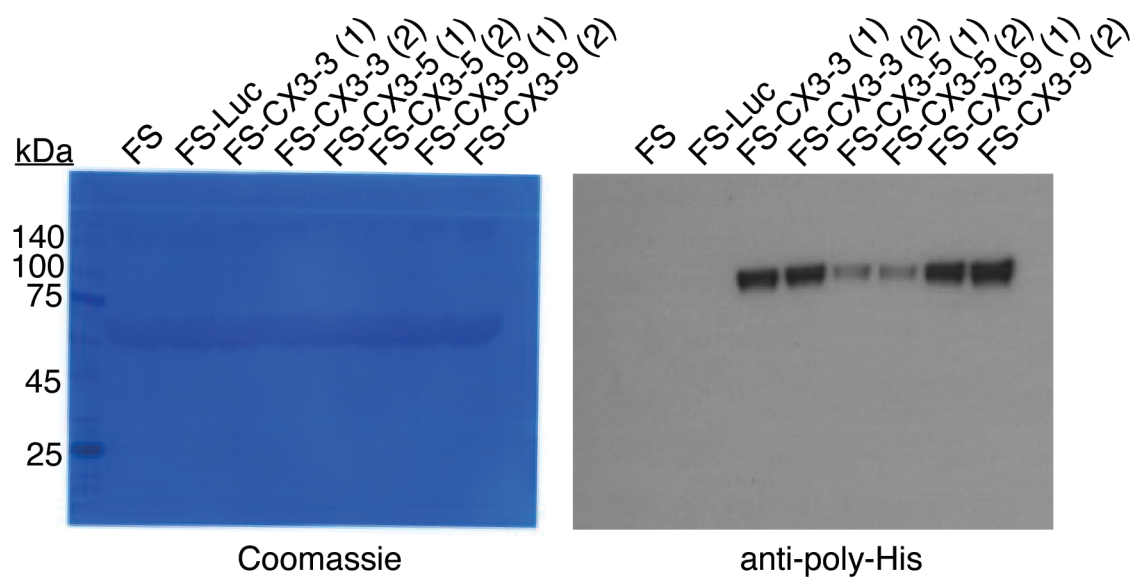

Supplement: Supplementary file 2 — Additional file 2. Fig. S1 Generation and characterization of sCX3CL1-expressing hESC. A Schematic of sCX3CL1 construct. B Plots representing the sort of top 4.72% mCherry-expressing FS hESC that were transfected with the sCX3CL1 piggyBac expression cassette. C Western blotting analysis against 8×-His tag of hESC conditioned media and respective global protein stain (n = 2–3). Glycine, G; Serine, S; FLAG, FLAG tag; Histidine, His. [file 13287_2023_3434_MOESM2_ESM.pdf]

**A**

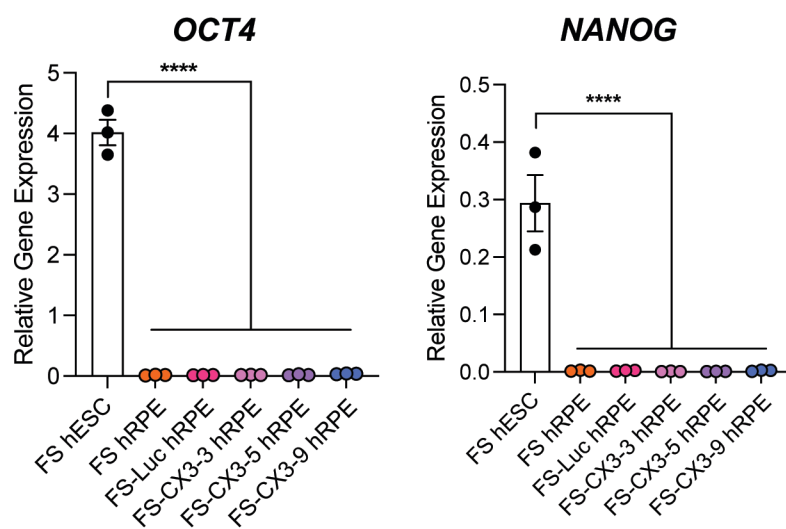

**B**

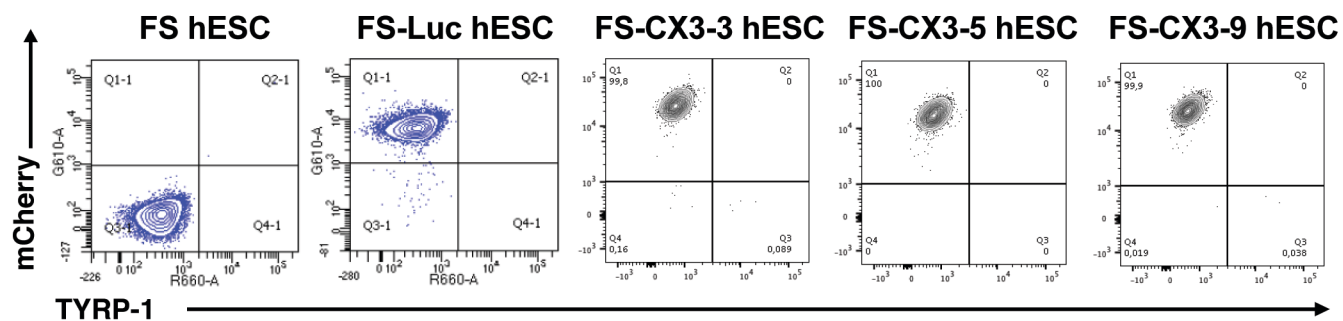

Supplement: Supplementary file 3 — Additional file 3. Fig. S2. Characterization of the expression of pluripotent stem cell markers in hRPE cells and the expression of a mature hRPE marker in hESC. A RT-qPCR against a panel of pluripotency markers (OCT4, NANOG). Gene expression normalized to GAPDH and expressed as relative to YWHAZ. B Representative flow cytometry analysis plots of TYRP-1 and mCherry expression in parental and transgenic hESC. Data analyzed by one-way ANOVA and Tukey’s post-hoc analysis. ****p < 0.0001. Fig. S3. Full-length western blotting of hRPE conditioned media. Western blotting analysis against 8×-His tag of hESC conditioned media and respective global protein stain (n = 2–3). [file 13287_2023_3434_MOESM3_ESM.pdf]

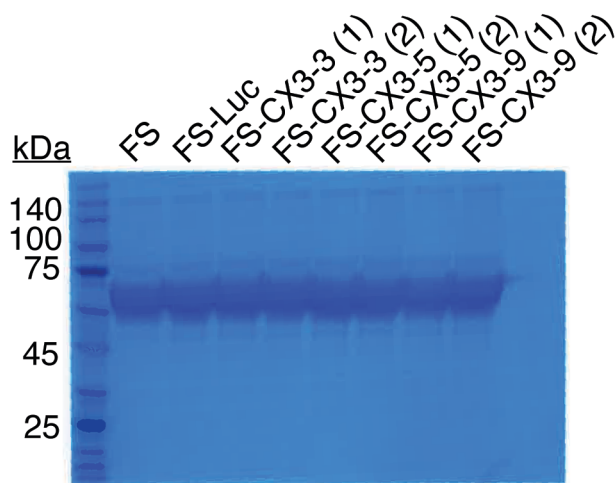

Coomassie

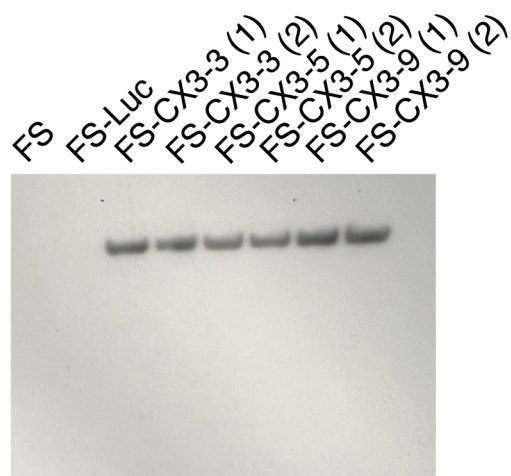

anti-poly-His

Supplement: Supplementary file 4 — Additional file 4. Fig. S3. Full-length western blotting of hRPE conditioned media. Western blotting analysis against 8×-His tag of hESC conditioned media and respective global protein stain (n = 2–3). [file 13287_2023_3434_MOESM4_ESM.pdf]

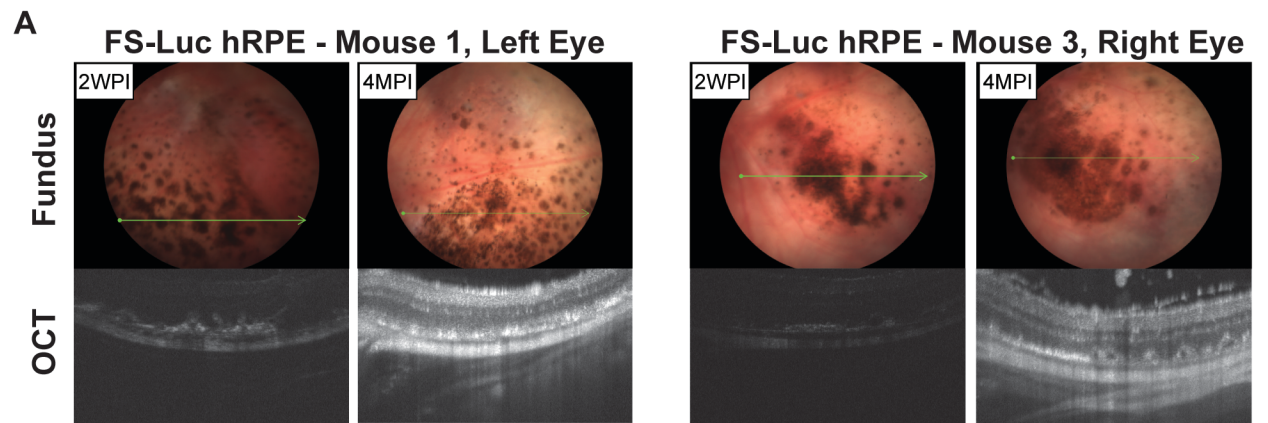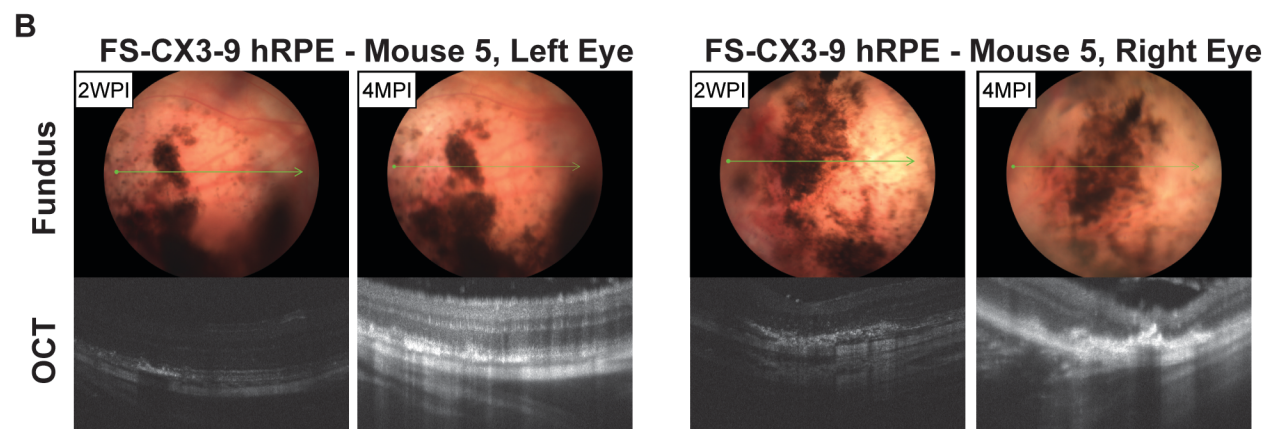

Supplement: Supplementary file 5 — Additional file 5. Fig. S4 In vivo imaging of NSG mice. Representative fundus and optical coherence tomography images (OCT) of A FS-Luc hRPE- or B FS-CX3-9 hRPE-treated NSG retinas. WPI, weeks post-injection; MPI, months post-injection. [file 13287_2023_3434_MOESM5_ESM.pdf]

**A**

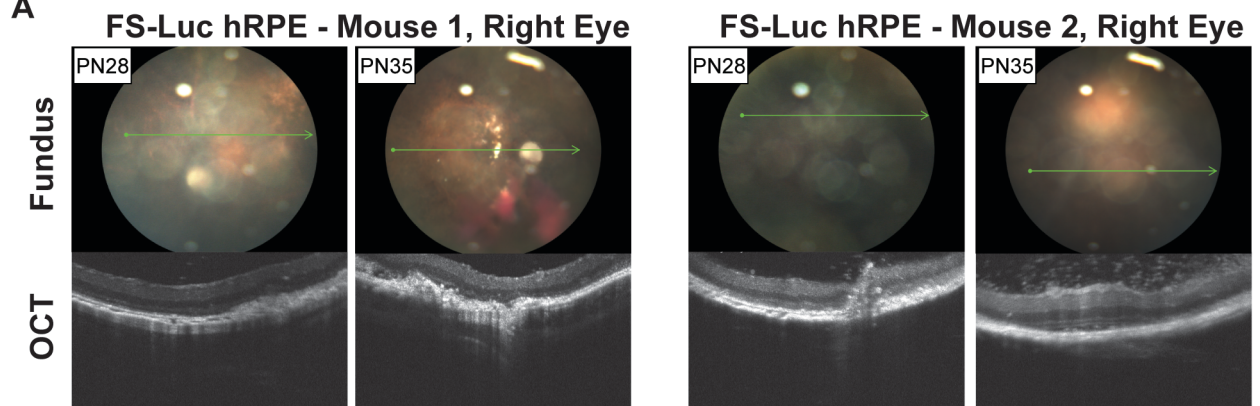

**B**

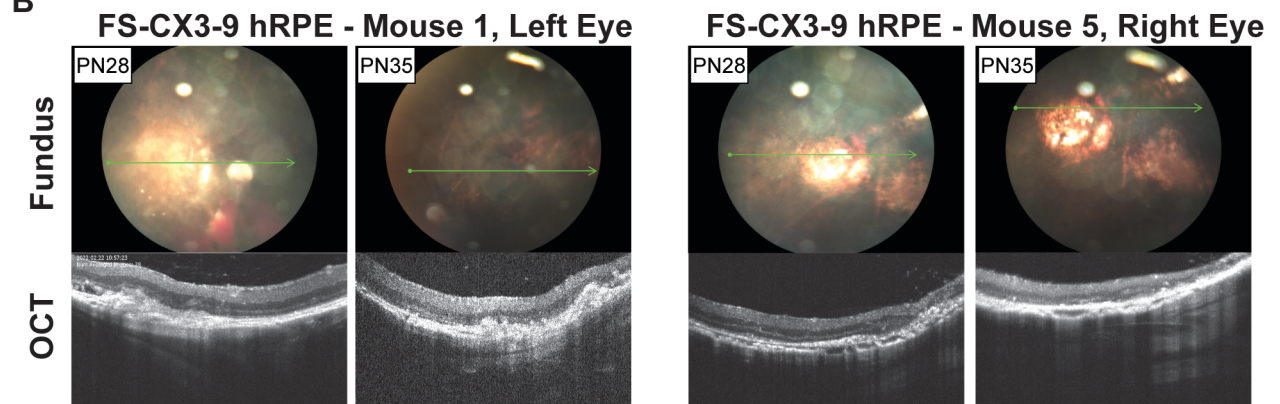

Supplement: Supplementary file 6 — Additional file 6. Fig. S5 In vivo imaging of rd10 mice. Representative fundus and optical coherence tomography images (OCT) of A FS-Luc hRPE- or B FS-CX3-9 hRPE-treated rd10 retinas. PN, postnatal. [file 13287_2023_3434_MOESM6_ESM.pdf]

NSG

**B6 +CsA**

**B6**

D2

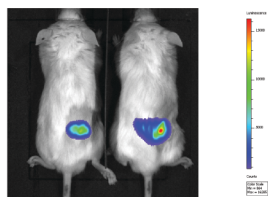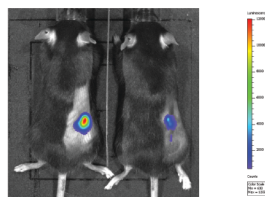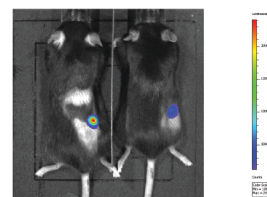

D4

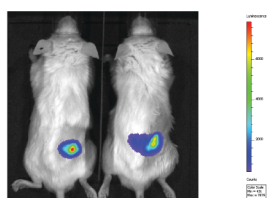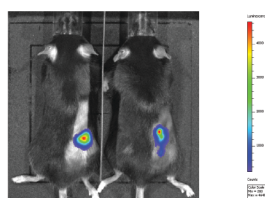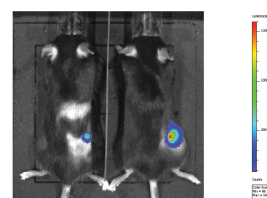

**D7**

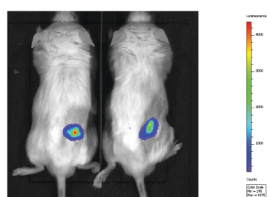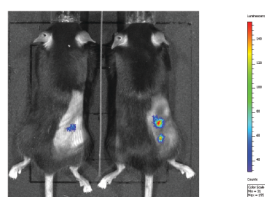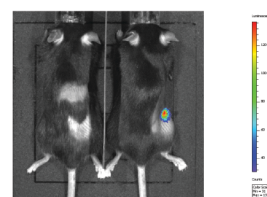

D9

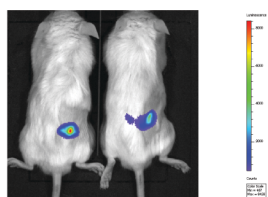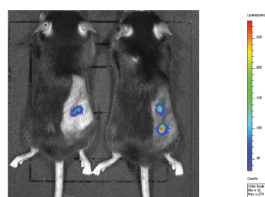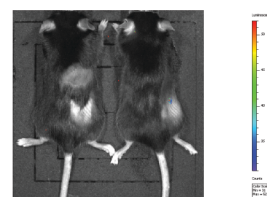

D11

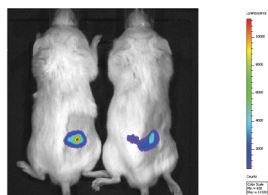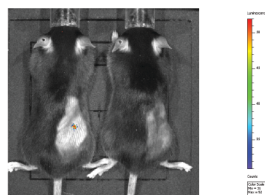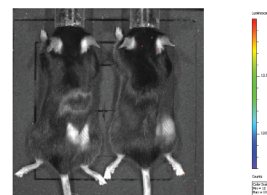

D14

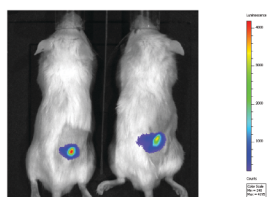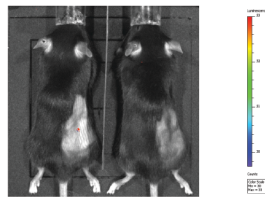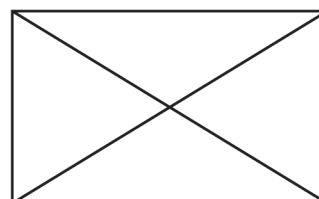

Supplement: Supplementary file 7 — Additional file 7. Fig. S6 Human hRPE delivered to flank subcutaneous are rejected in B6 mice. Representative bioluminescence imaging of mice treated with subcutaneous injections of FS-Luc hRPE at indicated timepoints. Cyclosporine A, CsA. (n = 3–4 mice). [file 13287_2023_3434_MOESM7_ESM.pdf]

A

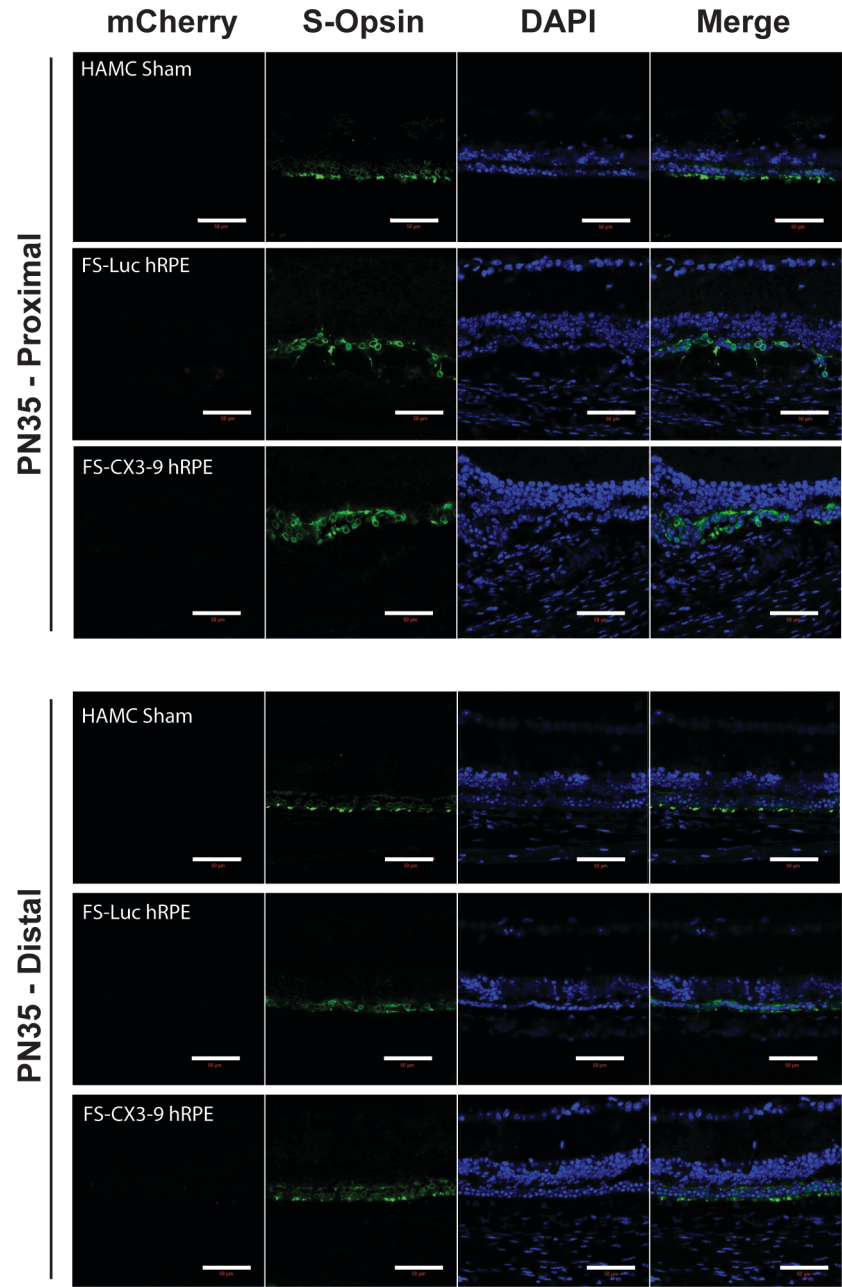

B

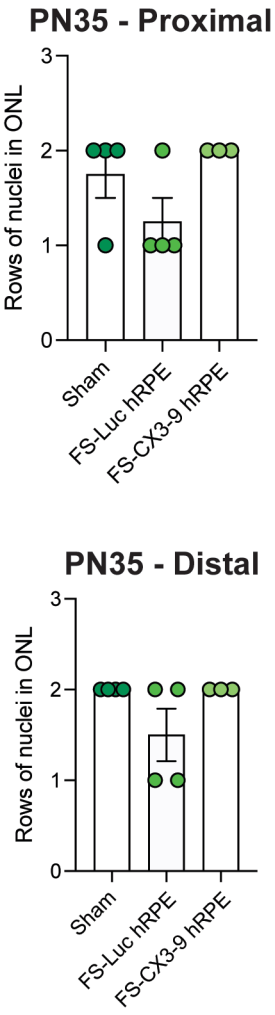

Supplement: Supplementary file 8 — Additional file 8. Fig. S7 Photoreceptor preservation in the rd10 mouse is not observed on PN35. Rd10 mice were treated on PN14, prior to the onset of rod degeneration, with subretinal injections of FS-Luc or FS-CX3-9 hRPE in HAMC, or HAMC alone (sham). Mice were sacrificed on PN35 for analysis. A Retinas immunostained for s-opsin. mCherry fluorescent reporter is expressed by injected cells. B Quantification of rows of nuclei (n = 3–4 eyes, each consisting of 3 technical replicates or tissue sections analyzed). Scale is 50 µm. One-way ANOVA and Tukey’s post-hoc analysis performed for statistical analysis. All comparisons are not statistically significant. ONL, outer nuclear layer; SRS, subretinal space. [file 13287_2023_3434_MOESM8_ESM.pdf]
